# Self-rated health and mental health before and during the early phase of the COVID-19 pandemic in Germany: the population-based German National Cohort (NAKO) study

**DOI:** 10.1186/s12889-026-27633-5

**Published:** 2026-05-09

**Authors:** Yue Xi, Thomas Keil, Lilian Krist, Tobias Pischon, Ilais Moreno Velásquez, Matthias B Schulze, Wolfgang Ahrens, Hajo Zeeb, Oliver Kuß, Tamara Schikowski, Börge Schmidt, Andreas Stang, Alexandra Nieters, Rafael Mikolajczyk, Janka Massag, Volker Harth, Nadia Obi, Anne C Hallet, Carolina J. Klett-Tammen, Wolfgang Lieb, Markus Löffler, Rudolf Kaaks, Till Bärnighausen, André Karch, Klaus Berger, Muhammad Nasir Khan Khattak, Claudia Meinke-Franze, Michael Leitzmann, Beate Fischer, Hermann Brenner, Bernd Holleczek, Susanne Rospleszcz, Annette Peters

**Affiliations:** 1 Institute of Epidemiology, Helmholtz Zentrum München - German Research Center for Environmental Health (GmbH), Ingolstädter Landstr. 1, Neuherberg, 85764 Germany; 2 Institute for Medical Information Processing, Biometry, and Epidemiology (IBE), Faculty of Medicine, LMU Munich, Pettenkofer School of Public Health, Munich, Germany; 3 Institute of Social Medicine, Epidemiology and Health Economics, Charité – Universitätsmedizin Berlin, Berlin, Germany; 4 Institute of Clinical Epidemiology and Biometry, University of Wuerzburg, Wuerzburg, Germany; 5 State Institute of Health I, Bavarian Health and Food Safety Authority, Erlangen, Germany; 6 Max Delbrück Center for Molecular Medicine in the Helmholtz Association (MDC), Molecular Epidemiology Research Group, Berlin, Germany; 7 Max Delbrück Center for Molecular Medicine in the Helmholtz Association (MDC), Biobank Technology Platform, Berlin, Germany; 8 Department of Molecular Epidemiology, German Institute of Human Nutrition Potsdam-Rehbruecke (DIfE), Nuthetal, Germany; 9 Institute of Nutritional Science, University of Potsdam, Nuthetal, Germany; 10 Leibniz Institute for Prevention Research and Epidemiology – BIPS, Bremen, Germany; 11 Health Sciences Bremen, University of Bremen, Bremen, Germany; 12 Institute for Biometrics and Epidemiology, German Diabetes Center, Leibniz Center for Diabetes Research at Heinrich Heine University, Düsseldorf, Germany; 13 Centre for Health and Society, Medical Faculty and University Hospital Düsseldorf, Heinrich Heine University Düsseldorf, Düsseldorf, Germany; 14 IUF - Leibniz Research Institute for Environmental Medicine, Düsseldorf, Germany; 15 Institute for Medical Informatics, Biometry and Epidemiology, University Hospital Essen, University of Duisburg-Essen, Essen, Germany; 16 Center for Chronic Immunodeficiency (CCI), University of Freiburg Medical Center, Freiburg, Germany; 17 Institute for Medical Epidemiology, Biometrics and Informatics, Medical Faculty of the Martin Luther University Halle-Wittenberg, Halle (Saale), Germany; 18 German Center for Mental Health (DZPG), partner site Halle-Jena-Magdeburg, Halle (Saale), Germany; 19 Institute for Occupational and Maritime Medicine Hamburg (ZfAM), University Medical Center Hamburg-Eppendorf (UKE), Hamburg, Germany; 20 Department of Epidemiology, Helmholtz Centre for Infection Research (HZI), Braunschweig, Germany; 21 Midwifery Research and Education Unit, Hannover Medical School, Braunschweig, Germany; 22 Institute of Epidemiology, Kiel University, Kiel, Germany; 23 Institute for Medical Informatics, Statistics and Epidemiology, University of Leipzig, Leipzig, Germany; 24 Division of Cancer Epidemiology, German Cancer Research Center (DKFZ), Heidelberg, Germany; 25 Heidelberg Institute of GlobalHealth, University Hospital Heidelberg, Heidelberg, Germany; 26 Institute of Epidemiology and Social Medicine, University of Münster, Münster, Germany; 27 Institute for Community Medicine, University Medicine Greifswald, Greifswald, Germany; 28 Institute for Epidemiology and Preventive Medicine, University of Regensburg, Regensburg, Germany; 29 Cancer Prevention Graduate School, German Cancer Research Center (DKFZ), Heidelberg, Germany; 30 Saarland Cancer Registry, Saarbrücken, Germany; 31 Department of Diagnostic and Interventional Radiology, Medical Center, Faculty of Medicine, University of Freiburg, Freiburg, Germany

**Keywords:** Self-rated health, Depression, Anxiety, Stress, COVID-19 pandemic

## Abstract

**Background:**

The COVID-19 pandemic and accompanying social distancing measures might have caused adverse health consequences. We aimed to describe changes in participants’ self-rated health and mental health (depression, anxiety, and stress), and investigate factors associated with them.

**Methods:**

We collected data from the German National Cohort (NAKO). We first described changes in participants’ self-rated health and mental health from the baseline examination (1 to 6 years earlier) to the early phase of the COVID-19 pandemic. We then applied the multinomial logistic regression model (self-rated health) and the quantile regression model (mental health) to investigate the potential factors associated with the health status and changes.

**Results:**

After a median of 3.1 [2.1, 4.1] years from baseline to the early pandemic phase (*N* = 91,809), 39.3% of participants with good health and 69.7% with less good health status at baseline reported better health. However, the percentage of participants with high depression, anxiety, and stress scores (≥ 10) increased from 6.2%, 4.1%, and 4.3% to 8.6%, 5.6%, and 10.1%, respectively. In the multivariable models, we found that being younger, being male, highly educated, being employed, having higher life satisfaction at baseline, being more physically active, drinking heavily, and experiencing improved anxiety symptoms were associated with improved self-rated health. In contrast, smoking and having mental health disorders were all associated with worse self-rated health. Our results showed that being younger, being female, smoking, drinking heavily, and drinking more since baseline were associated with higher depression scores. Having had a coronavirus test was associated with worse self-rated health and more severe anxiety and stress.

**Conclusions:**

During the early COVID-19 pandemic, many participants experienced improvements in self-rated health but suffered deterioration in mental health and physical activity engagement. Female participants, those who were physically inactive, and those with pre-existing mental disorders were more likely to report poorer health.

**Supplementary Information:**

The online version contains supplementary material available at 10.1186/s12889-026-27633-5.

## Background

The Coronavirus Disease 2019 (COVID-19) pandemic has unprecedentedly affected global health. Governments around the world have implemented nationwide lockdowns, accompanied by several comprehensive measures, which included mandatory closure of educational institutions, recommendations for working from home for employees whenever possible, regulations and prohibitions on mass gatherings, self-isolation, quarantine in case of exposure or infection, and limitation of non-essential services, to prevent the spread of COVID-19. In Germany, six-week nationwide countermeasures were introduced two weeks after the confirmation of the first case to limit the spread of COVID-19 and safeguard the general population [1]. However, concerns have been raised regarding the potential health consequences of such social distancing measures: individuals were isolated owing to the protective measures, accompanied by restrictions on daily activities that influence health behaviors such as cigarette smoking, alcohol consumption, and physical activity [2, 3]. The COVID-19 pandemic, social isolation, and behavior changes resulting from the protective measures all affected health.

A large number of studies have described the mental health status [4, 5] and physical health [6–11] during the COVID-19 pandemic. Large heterogeneity was found across different populations, so research based on well-powered studies from different populations is still needed. Interestingly, current evidence shows that mental health changes with the process of the pandemic: the mental health scores increased (indicating severe symptoms) at the beginning of the pandemic and then decreased [5, 12]. Based on data from the German population, Peters et al. [6] reported that 32% of the participants stated an improvement in their self-rated health from baseline to the early pandemic (May 2020) when a COVID-NAKO survey was conducted, while participants’ mean scores for self-perceived stress, depression, and anxiety increased. However, changes in self-rated health among participants with specific self-rated health status at baseline, and the percentages of participants with deteriorated mental health above clinically meaningful levels, are not yet described.

As health status is complex and multi-faceted, many studies have identified populations’ characteristics concerning self-rated health and mental health during the pandemic, such as age, sex, socioeconomic status, activity, and pre-existing health conditions [9–15]. In the former study conducted by Peters and colleagues, they examined the associations between age, sex, self-rated health status at baseline, and coronavirus testing with changes in self-rated health and mental health [6]. However, the co-effects of other factors, e.g., financial status, cohabitation, education, lifestyle (smoking, alcohol consumption, and physical activity), and other health aspects, were not taken into account. A comprehensive analysis of all these factors simultaneously might help to identify individuals at high risk of impaired mental and general health during the pandemic, as well as provide clues for potential preventive measures.

Therefore, this study aimed to (1) describe changes in participants’ self-rated health and mental health (depression, anxiety, and stress) between the baseline examination and the early phase of the COVID-19 pandemic; (2) investigate factors associated with the health status during the pandemic and health changes from pre-pandemic to the pandemic.

## Methods

### Study population

The German National Cohort (NAKO, NAKO Gesundheitsstudie) is a large, multidisciplinary, prospective population-based cohort. From 2014 to 2019, a total of 205,415 men and women aged 20 to 74 years who were randomly recruited from 18 study centers across Germany participated in the baseline examinations. More detailed information on the NAKO study design and methods can be found elsewhere [16, 17]. To collect information regarding the COVID-19 pandemic and nationwide protective countermeasures, a dedicated COVID-NAKO questionnaire was implemented on 30th April 2020. The current study is based on data from participants who completed the COVID-NAKO questionnaires before 29th May, which accounts for 56.1% of the total cohort sample size. We included 91,809 participants for whom data from before the pandemic (baseline) and during the first wave of the pandemic were available in the analysis, after excluding participants with missing values (Fig. S1 & Table S1).

The NAKO was approved by all study centers’ local ethics committees, and all participants provided written consent for study participation and repeated follow-up.

### Variable assessment

#### Self-rated health and mental health

We assessed self-rated health and mental health at the baseline examination and in the COVID-NAKO survey. Self-rated health from the Short Form Health Questionnaire (SF-12) was measured on a 5-point Likert scale: “Excellent”, “Very Good”, “Good”, “Less Good”, and “Bad”. We combined the “Less Good” and “Bad” groups for health status analysis. Several modules from the German version of the Patient Health Questionnaire (PHQ) were included in the questionnaires to assess mental health: depressive symptoms (PHQ-9), anxiety symptoms (GAD-7), and perceived psychosocial strains (PHQ-stress) [18, 19]. A higher score indicates more severe mental health symptoms.

Differences from the baseline examination to the COVID-NAKO questionnaire were calculated for all participants with data available at both time points. Based on the calculated changes, we collapsed the differences into three categories: “Better” (lower scores during the pandemic), “Same” (same scores), and “Worse” (higher scores) for further analysis.

#### Other factors

All of the potential associated factors were collected from either the baseline or COVID-NAKO questionnaires. Demographic variables: age at baseline examination, sex, education status at baseline, employment status, cohabitation, and change in household financial status in the COVID-NAKO questionnaire. Lifestyle factors: cigarette smoking status, and alcohol consumption frequency at baseline examination and in the COVID-NAKO questionnaire, as well as changes in physical activity and sitting behaviors in the COVID-NAKO questionnaire. Life satisfaction at baseline examination. COVID-19-related variables: having had a coronavirus test during the early phase of the pandemic.

### Statistical analysis

We first described participants’ characteristics stratified by surveys (baseline examination, COVID-NAKO). For the variables with two measurements, we applied the Wilcoxon Signed-rank Test/Pearson’s Chi-squared Test to examine the difference between the two surveys and drew Sankey plots to show their changes between the baseline examination and the COVID-NAKO questionnaire. We then summarized the percentages of different health changes for both self-rated health and mental health.

To identify factors associated with self-rated health status during the pandemic and its changes from the baseline examination to the COVID-NAKO questionnaire, we fitted multinomial logistic regression models and calculated the relative risk ratio (RRR) and 95% confidence interval (CI). For mental health scales and their changes, we applied linear and quantile regression to examine potentially associated factors. The quantile regression is a robust alternative to linear regression, in which we estimated the effects of predictors at multiple quantile levels (0.10, 0.25, 0.50, 0.75, and 0.90). We calculated the adjusted *P* value with Bonferroni correction due to the simultaneous multiple testing. More details are presented in the supplementary materials.

### Sensitivity analysis

To evaluate the robustness of the results, we performed several sensitivity analyses. We first adjusted for the baseline examination year or the date of the COVID-NAKO survey to control potential temporal trends. We then included study centers as additional covariates to account for geographic heterogeneity. Finally, to mitigate potential selection bias, we estimated inclusion probabilities based on baseline age and sex and incorporated inverse probability weighting into the regression analyses.

All analyses were performed with R statistical software, version 4.3.1.

## Results

Table 1 shows participants’ characteristics obtained in both surveys. A total of 91,809 participants were included in the current study, with a mean age of 48.8 (12.4) years, and 46,574 (50.7%) were women. Among the participants, 4,001 (4.4%), 31,907 (34.8%), 48,544 (52.9%), and 7,357 (8.0%) reported “Excellent”, “Very Good”, “Good”, and “Less Good or Bad” for self-rated health status, respectively, at baseline examination. After a median of 3.1 [2.1, 4.1] years, 9,194 (10.0%), 39,695 (43.2%), 37,554 (40.9%), and 5,366 (5.8%) participants rated their health as “Excellent”, “Very Good”, “Good”, and “Less Good or Bad”, respectively. Meanwhile, their median depression, anxiety, and stress scores changed from 3.0, 2.0, and 3.0 at baseline to 3.0, 3.0, and 4.0, respectively, during the pandemic. We also found that 6.2%, 4.1%, and 4.3% of participants had high depression, anxiety, and stress scores (≥ 10) at baseline examination, which increased to 8.6%, 5.6%, and 10.1%, respectively, during the COVID-NAKO survey.

**Table 1:** Characteristics of study participants (*N* = 91,809)

Variables	Baseline (2014–2019)	COVID-NAKO (May 2020)	*P* value*
Age (years), Mean (SD)	48.8 (12.4)		
Gender: Male	45,235 (49.3%)		
Education at baseline: University	46,278 (50.4%)		
Employment status: Employed		67,721 (73.8%)	
Cohabitation: Living with others		76,806 (83.7%)	
Financial status		3,212 (3.5%)	
Same		69,512 (75.7%)	
Deteriorated		19,085 (20.8%)	
Improved		3,212 (3.5%)	
Household physical activity			
Same		55,908 (60.9%)	
Less		4,164 (4.5%)	
More		31,737 (34.6%)	
Sport-related physical activity			
Same		35,025 (38.1%)	
Less		39,472 (43.0%)	
More		17,312 (18.9%)	
Sitting behavior			
Same		56,809 (61.9%)	
Less		6,845 (7.5%)	
More		28,155 (30.7%)	
Smoking status			< 0.001
Never	45,117 (49.1%)	48,811 (53.2%)	
Past	31,327 (34.1%)	30,203 (32.9%)	
Current	15,365 (16.7%)	12,795 (13.9%)	
Alcohol consumption			< 0.001
Never	6,074 (6.6%)	13,316 (14.5%)	
≤ 1 time/month	15,455 (16.8%)	11,195 (12.2%)	
2–4 times/month	28,067 (30.6%)	22,452 (24.5%)	
2–4 times/week	24,949 (27.2%)	28,655 (31.2%)	
5–6 times/week	12,187 (13.3%)	10,138 (11.0%)	
Everyday	5,077 (5.5%)	6,053 (6.6%)	
Life satisfaction, Median [IQR]	8.0 [7.0, 9.0]		
Having had a coronavirus test		4354 (4.7%)	
Self-rated health status			< 0.001
Excellent	4,001 (4.4%)	9,194 (10.0%)	
Very good	31,907 (34.8%)	39,695 (43.2%)	
Good	48,544 (52.9%)	37,554 (40.9%)	
Less good or bad	7,357 (8.0%)	5,366 (5.8%)	
Depression score, Median [IQR]	3.0 [1.0, 5.0]	3.0 [1.0, 6.0]	< 0.001
≥10	5,679 (6.2%)	7,925 (8.6%)	< 0.001
Anxiety score, Median [IQR]	2.0 [1.0, 4.0]	3.0 [1.0, 5.0]	< 0.001
≥10	3,785 (4.1%)	5,168 (5.6%)	< 0.001
Stress score, Median [IQR]	3.0 [1.0, 5.0]	4.0 [2.0, 7.0]	< 0.001
≥10	3,920 (4.3%)	9,283 (10.1%)	< 0.001
Time since baseline examination (years), Median [IQR]	3.1 [2.1, 4.1]		

For continuous variables with normal distribution, we present Mean (SD) and Median [IQR] for those without normal distribution. For categorical variables, we present numbers (%)

*For the variables with two measurements, including cigarette smoking status, alcohol consumption frequency, self-rated health, and mental health symptoms (binary variables and scores), we applied the Wilcoxon Signed-rank Test/Pearson’s Chi-squared Test to examine the difference between baseline examination and the COVID-NAKO survey

**Fig. 1:**
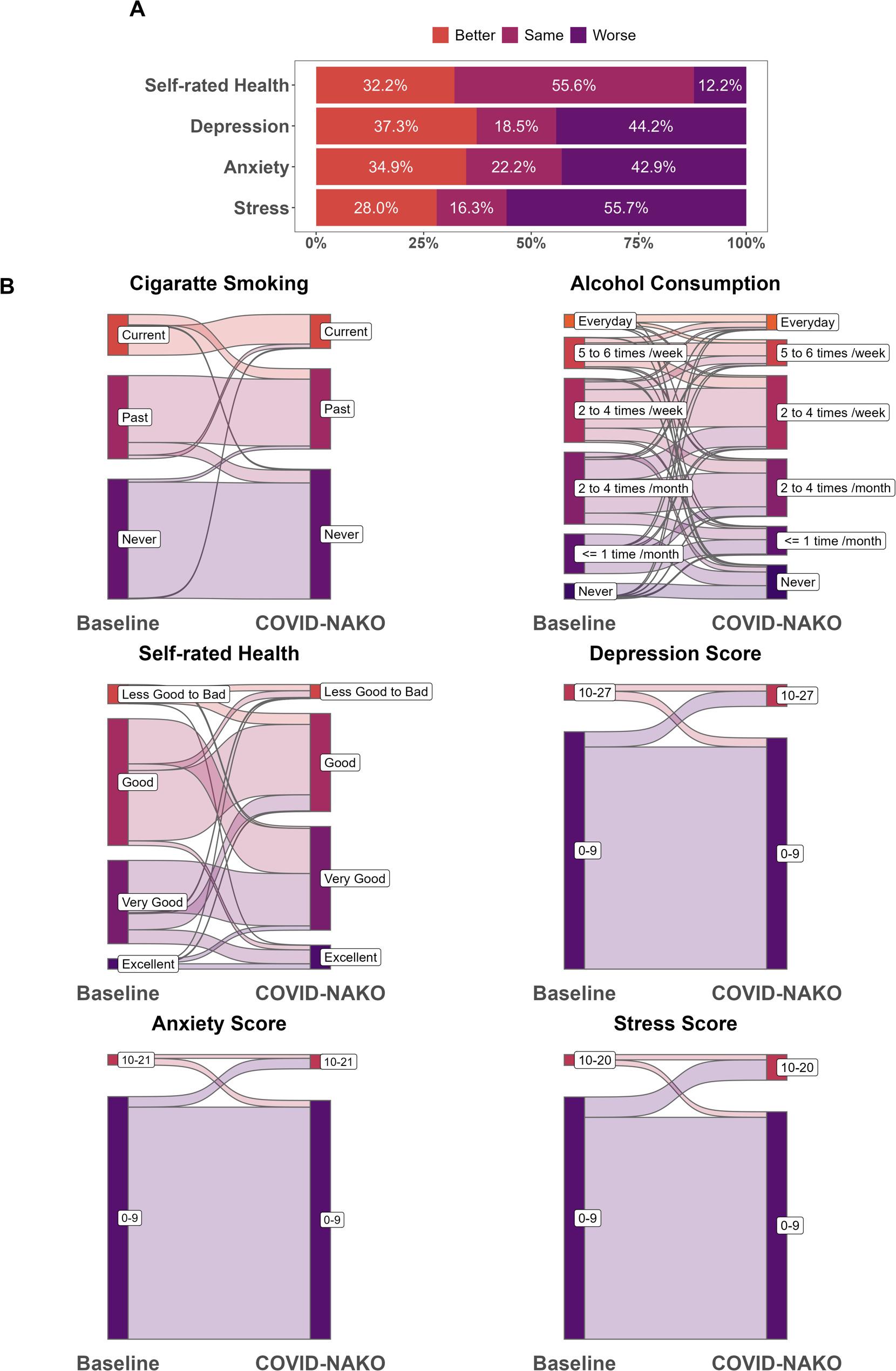
Descriptive statistics. **A** The percentage of health changes. **B** Participants’ migration from baseline examination (2014-2019) to COVID-NAKO survey (May 2022)

Figure 1A is about the components of health changes from baseline to the COVID-NAKO survey. It shows that 33.2% of the participants experienced improved self-rated health, while 12.2% suffered deteriorated self-rated health. For mental health, 44.2%, 42.9%, and 55.7% of the participants had higher scores, while 37.3%, 34.9%, and 28.0% had lower scores for depression, anxiety, and stress scores, respectively. Figure 1B presents the changes between the two surveys. For smoking, most of the participants remained in the same groups, but the frequency of alcohol consumption tended to change for many people. Most of the participants remained in the same groups for self-rated health, but we found that 39.3% of participants with good health at baseline moved to better health status during the pandemic, as did 69.7% of participants with less good health. Some of our participants experienced improved mental health, but more people suffered deteriorated mental health changes.

### The factors associated with self-rated health

Table 2 shows the results of factors associated with change in self-rated health from the multinomial logistic model. We found that being male, having high education, being employed, having deteriorated financial status, having high life satisfaction at baseline, having more physical activity, drinking heavily during the pandemic, and experiencing improved anxiety symptoms from baseline to the pandemic were associated with “Better” self-rated health. In contrast, being older, having had a coronavirus test, being less physically active, having more sitting behaviors, smoking during the pandemic, having mental health disorders during the pandemic, quitting smoking, and drinking less alcohol from baseline to COVID-NAKO were all associated with “Worse” self-rated health. For self-rated health status during the pandemic, we found similar results to those for health change (Table S1).

**Table 2:** Factors associated with self-rated health change from baseline to early COVID-19

Variables	Better	Worse
Age at baseline (years)	0.97(0.97,0.97)*	1.02(1.01,1.02)*
Gender: Male	1.15(1.11,1.19)*	0.90(0.86,0.94)*
Education at baseline: University	1.11(1.08,1.15)*	0.85(0.81,0.89)*
Employment status: Employed	1.10(1.05,1.14)*	0.88(0.83,0.93)*
Cohabitation: Living with others	0.96(0.92,1.00)	0.99(0.93,1.05)
Financial status: Deteriorated	1.09(1.05,1.13)*	0.95(0.90,1.01)
Financial status: Improved	1.07(0.98,1.16)	0.94(0.83,1.06)
Life satisfaction at baseline	1.12(1.11,1.13)*	0.94(0.93,0.95)*
Coronavirus test	0.78(0.72,0.84)*	1.60(1.46,1.75)*
Household physical activity: Less	0.85(0.78,0.92)*	1.44(1.31,1.59)*
Household physical activity: More	1.05(1.01,1.09)*	0.97(0.93,1.02)
Sport-related physical activity: Less	0.96(0.93,1.00)	1.14(1.09,1.20)*
Sport-related physical activity: More	1.37(1.31,1.43)*	0.78(0.73,0.83)*
Sitting behavior: Less	1.02(0.96,1.09)	1.11(1.02,1.21)
Sitting behavior: More	0.90(0.87,0.93)*	1.13(1.08,1.19)*
Smoking status: Past smoker	0.96(0.93,1.00)	1.14(1.09,1.20)*
Smoking status: Current smoker	0.90(0.85,0.94)*	1.23(1.14,1.31)*
Alcohol consumption: 2 to 4 times/month	1.03(0.98,1.07)	0.86(0.81,0.92)*
Alcohol consumption: 2 to 4 times/week	1.11(1.06,1.15)*	0.78(0.73,0.83)*
Alcohol consumption: ≥ 5 times/week	1.12(1.07,1.18)*	0.82(0.77,0.88)*
Depression score: ≥10	0.53(0.50,0.57)*	2.32(2.13,2.52)*
Anxiety score: ≥10	0.77(0.71,0.84)*	1.64(1.49,1.81)*
Stress score: ≥10	0.64(0.60,0.68)*	1.79(1.66,1.92)*
Smoking change: Yes to No^b^	0.98(0.91,1.05)	1.22(1.10,1.35)*
Smoking change: No to Yes^b^	1.00(0.89,1.12)	1.18(1.01,1.38)
Smoking change: Remained Yes^b^	0.90(0.86,0.95)*	1.17(1.09,1.25)*
Alcohol consumption change: Less^b^	0.96(0.92,0.99)	1.15(1.09,1.21)*
Alcohol consumption change: More^b^	1.00(0.96,1.04)	0.93(0.88,0.98)
Depression change: Decreased from ≥ 10 to < 10^b^	1.02(0.93,1.12)	1.14(0.96,1.35)
Depression change: Increased from < 10 to ≥ 10^b^	0.53(0.48,0.57)*	2.42(2.21,2.65)*
Depression change: Remained ≥ 10^b^	0.53(0.47,0.60)*	2.08(1.76,2.45)*
Anxiety change: Decreased from ≥ 10 to < 10^b^	1.24(1.11,1.38)*	0.98(0.81,1.18)
Anxiety change: Increased from < 10 to ≥ 10^b^	0.79(0.71,0.87)*	1.66(1.50,1.84)*
Anxiety change: Remained ≥ 10^b^	0.81(0.69,0.95)	1.63(1.34,1.99)*
Stress change: Decreased from ≥ 10 to < 10^b^	0.86(0.78,0.96)	1.21(1.00,1.46)
Stress change: Increased from < 10 to ≥ 10^b^	0.62(0.58,0.66)*	1.83(1.69,1.98)*
Stress change: Remained ≥ 10^b^	0.66(0.59,0.75)*	1.70(1.44,2.01)*

The table presents the Relative Risk Ratio (RRR) and 95% confidence interval (CI) from the multinomial logistic regression model, using “Same” as the reference group

The model included smoking, alcohol, and mental health scales during COVID-19 and was adjusted with health status at baseline and time from baseline to the COVID-NAKO survey

^b^The model included variables’ changes from baseline to the COVID-NAKO survey. *Statistically significant after Bonferroni correction

### The factors associated with mental health

Table 3 shows the results of factors associated with depression scores in the COVID-NAKO questionnaire. Our results showed that having high education, having deteriorated financial status, having poor self-rated health, having changed physical activity and sitting behaviors, smoking, drinking heavily during COVID-19, drinking more since baseline, and having other mental scales during the pandemic were all associated with higher depression scores. Being older, being male, being employed, living with others, and having high life satisfaction at baseline were associated with lower depression scores. Table S2 and Table S3 show the results for anxiety and stress. In contrast to depression, being employed, living with others, and having had a coronavirus test were associated with more severe anxiety and stress. We also found that life satisfaction was not associated with anxiety but with increased stress scores, while self-rated health was not associated with stress. Having higher education was associated with increased stress scores in individuals with initially low scores, but decreased scores in people with high scores. The results for changes in mental health were very similar to those for mental health status (Table S4 - S6).

**Table 3:** Factors associated with the depression score during early COVID-19

Variables	Linear	Q10	Q25	Q50	Q75	Q90
Age at baseline (years)	-0.04(-0.05,-0.04)*	-0.02(-0.02,-0.01)*	-0.03(-0.03,-0.03)*	-0.04(-0.04,-0.04)*	-0.05(-0.06,-0.05)*	-0.06(-0.07,-0.06)*
Gender: Male	-0.33(-0.36,-0.29)*	-0.12(-0.13,-0.10)*	-0.24(-0.27,-0.21)*	-0.38(-0.41,-0.34)*	-0.44(-0.49,-0.39)*	-0.48(-0.56,-0.41)*
Education at baseline: University	0.16(0.12,0.19)*	0.09(0.08,0.10)*	0.15(0.11,0.18)*	0.16(0.12,0.20)*	0.17(0.12,0.23)*	0.16(0.08,0.23)*
Employment status: Employed	-0.10(-0.15,-0.06)*	-0.03(-0.05,-0.01)*	-0.01(-0.05,0.03)	-0.01(-0.06,0.04)	-0.08(-0.14,-0.01)	-0.16(-0.26,-0.07)*
Cohabitation: Living with others	-0.44(-0.49,-0.40)*	-0.11(-0.14,-0.09)*	-0.22(-0.26,-0.17)*	-0.30(-0.35,-0.25)*	-0.45(-0.53,-0.37)*	-0.73(-0.83,-0.62)*
Financial status: Deteriorated	0.51(0.47,0.56)*	0.16(0.13,0.18)*	0.30(0.25,0.34)*	0.41(0.36,0.47)*	0.68(0.61,0.76)*	0.86(0.76,0.96)*
Financial status: Improved	-0.10(-0.20,-0.01)	0.02(-0.03,0.06)	-0.09(-0.18,0.01)	-0.13(-0.22,-0.03)	-0.08(-0.21,0.06)	-0.06(-0.23,0.11)
Self-rated health status	0.84(0.81,0.87)*	0.28(0.26,0.29)*	0.54(0.52,0.56)*	0.76(0.74,0.79)*	0.95(0.92,0.99)*	1.11(1.05,1.16)*
Life satisfaction at baseline	-0.04(-0.05,-0.03)*	-0.01(-0.02,-0.01)*	-0.03(-0.04,-0.02)*	-0.04(-0.05,-0.03)*	-0.06(-0.08,-0.04)*	-0.06(-0.08,-0.04)*
Coronavirus test	-0.01(-0.09,0.07)	0.04(-0.00,0.09)	0.03(-0.05,0.11)	0.01(-0.07,0.09)	0.02(-0.10,0.13)	-0.01(-0.18,0.16)
Household physical activity: Less	1.49(1.40,1.57)*	0.87(0.69,1.04)*	1.24(1.13,1.35)*	1.50(1.39,1.62)*	1.78(1.64,1.93)*	2.01(1.72,2.30)*
Household physical activity: More	0.23(0.19,0.27)*	0.13(0.11,0.15)*	0.21(0.17,0.25)*	0.25(0.21,0.29)*	0.32(0.26,0.37)*	0.28(0.20,0.36)*
Sport-related physical activity: Less	0.39(0.35,0.43)*	0.16(0.15,0.17)*	0.27(0.24,0.31)*	0.36(0.31,0.40)*	0.45(0.40,0.51)*	0.50(0.42,0.59)*
Sport-related physical activity: More	0.12(0.07,0.17)*	0.07(0.05,0.08)*	0.09(0.05,0.13)*	0.14(0.09,0.19)*	0.17(0.10,0.24)*	0.17(0.06,0.27)*
Sitting behavior: Less	0.23(0.16,0.30)*	-0.00(-0.03,0.02)	0.09(0.03,0.15)*	0.20(0.12,0.29)*	0.34(0.24,0.45)*	0.38(0.24,0.53)*
Sitting behavior: More	0.49(0.45,0.53)*	0.23(0.20,0.25)*	0.38(0.35,0.42)*	0.49(0.44,0.53)*	0.57(0.51,0.63)*	0.64(0.56,0.73)*
Smoking status: Past smoker	0.11(0.07,0.14)*	0.05(0.04,0.06)*	0.07(0.03,0.10)*	0.10(0.06,0.14)*	0.15(0.10,0.21)*	0.18(0.10,0.26)*
Smoking status: Current smoker	0.11(0.06,0.17)*	0.03(0.01,0.05)*	0.01(-0.04,0.06)	0.08(0.02,0.15)	0.21(0.13,0.29)*	0.20(0.08,0.32)*
Alcohol consumption: 2 to 4 times/month	0.00(-0.04,0.05)	0.07(0.05,0.09)*	0.08(0.04,0.12)*	0.05(-0.00,0.10)	-0.03(-0.10,0.04)	-0.16(-0.27,-0.06)*
Alcohol consumption: 2 to 4 times/week	0.10(0.05,0.14)*	0.10(0.09,0.12)*	0.14(0.09,0.18)*	0.16(0.11,0.21)*	0.08(0.01,0.15)	-0.07(-0.18,0.03)
Alcohol consumption: ≥ 5 times/week	0.24(0.18,0.29)*	0.13(0.11,0.14)*	0.17(0.12,0.22)*	0.21(0.15,0.27)*	0.23(0.15,0.31)*	0.23(0.11,0.35)*
Anxiety score: ≥10	5.80(5.72,5.88)*	4.81(4.69,4.94)*	5.03(4.90,5.17)*	5.62(5.47,5.77)*	6.53(6.36,6.70)*	7.20(6.96,7.45)*
Stress score: ≥10	2.09(2.03,2.15)*	1.65(1.59,1.71)*	1.90(1.81,1.98)*	2.14(2.06,2.23)*	2.32(2.20,2.43)*	2.50(2.32,2.68)*
Smoking change: Yes to No^b^	0.11(0.02,0.19)	0.00(-0.04,0.05)	0.06(-0.02,0.13)	0.18(0.08,0.28)*	0.14(0.02,0.26)	0.22(0.01,0.42)
Smoking change: No to Yes^b^	0.20(0.07,0.32)*	0.05(0.01,0.08)*	0.03(-0.09,0.15)	0.13(-0.05,0.30)	0.26(0.08,0.44)*	0.23(0.02,0.44)
Smoking change: Remained Yes^b^	0.08(0.02,0.13)*	0.01(-0.02,0.03)	-0.00(-0.05,0.05)	0.05(-0.02,0.11)	0.17(0.08,0.25)*	0.15(0.04,0.26)
Alcohol consumption change: Less^b^	-0.05(-0.10,-0.01)	-0.04(-0.06,-0.03)*	-0.08(-0.11,-0.04)*	-0.05(-0.09,-0.01)	-0.01(-0.07,0.05)	-0.00(-0.09,0.08)
Alcohol consumption change: More^b^	0.22(0.17,0.26)*	0.06(0.04,0.08)*	0.10(0.06,0.14)*	0.16(0.11,0.20)*	0.24(0.18,0.31)*	0.35(0.26,0.44)*

The table presents the absolute changes in depression scale and 95% CI from the linear/quantile regression model

The model included smoking, alcohol, and mental health scales during COVID-19 and was adjusted with depression score at baseline and time from baseline to the COVID-NAKO survey

^b^The model included variables’ changes from baseline to the COVID-NAKO survey. *Statistically significant after Bonferroni correction

Results from the sensitivity analyses were very similar to those from the main analyses (Table S7 - S14).

## Discussions

In summary, the majority of participants did not report an extreme (excellent or very poor) self-rated health status, and we surprisingly found an improvement in self-rated health from baseline to early in the pandemic. In contrast, most participants reported deteriorated changes in mental health, particularly in stress, for which more than half of the participants experienced increased scores. In the multivariable regressions, we found that self-rated health and mental health were positively associated with each other and that being male was associated with better self-rated and mental health. Interestingly, our results showed that being older was associated with worse self-rated health but better mental health. Furthermore, our analyses showed that having had a coronavirus test was associated with worse self-rated health, anxiety, and stress, but not depression, while living with others was associated with lower depression but higher anxiety and stress scales. Overall, our findings suggest that certain populations have experienced more negative effects from COVID-19 and related countermeasures. It also highlights the need for public health interventions to protect populations from poorer health in a corresponding situation.

In the current population-based study, the lower mean of the self-rated health during the pandemic compared to baseline and a higher percentage of better change (32%) may indicate an improvement in self-rated health. At the same time, the higher mean of mental health scores and higher percentage of worse changes in depression, anxiety, and stress symptoms indicate more severe mental health symptoms. However, due to the lack of information on changes in health from baseline to pre-pandemic makes it difficult to draw causal inferences. Several studies investigated the self-rated health status during COVID-19 and found that although the impact of the pandemic on health may vary widely among different population groups, most people did not report extreme health (excellent or very poor), which is consistent with our findings [8, 9]. Our study reported an improvement in self-rated health from baseline to the early phase of the pandemic. Similar to this, a former research reported increased change in self-rated physical health from recruitment to the first lockdown among 1,237 French people [20]. We found that being younger was associated with improvement in self-reported health but deterioration in mental health. The age distribution (relatively younger) might contribute to the results. Furthermore, we found that lower physical activity was associated with worse self-rated health, whereas higher physical activity was associated with better self-rated health. However, both of them were associated with worse mental health. The relatively stronger associations with more physical activity, compared with less, might be another reason. However, most other studies reported deteriorated self-rated health, including in the U.S [10]., Scotland [13], Germany [21], Korea [7], and Japan [11]. Although there were inconsistencies between these different populations, most of the current studies found that people suffered health deterioration during the pandemic. The difference in population characteristics and the measurement windows from baseline to the pandemic may contribute to the difference between our findings and the previous research. Our study describes the health change from the baseline examinations (3.1 median years before) to the pandemic (after the lockdown). We cannot conclude that the health changes were caused by the pandemic and the corresponding countermeasures, in part because of the lack of information on the health status before COVID-19.

Participants in our study reported more severe mental health symptoms during the COVID-19 pandemic compared with their status at baseline. Evidence showed that participants’ mental health changed as the pandemic progressed and the protective measures were implemented. A meta-analysis of 43 records (*N* = 71,613 participants) found that both anxiety and depression were increased during the early phase of the pandemic when compared with the pre-pandemic period [22]. A systematic review and meta-analysis of 65 longitudinal cohort studies observed an early increase in overall mental health symptoms compared with the pre-pandemic status, which subsequently declined over time and became non-significant, but the increase in depression tended to be larger and remained significant in the later period [12]. In a meta-analysis including a total of 331,628 participants from 43 studies, depression and anxiety symptoms worsened in the first 2 months of the pandemic; thereafter, the trajectories were heterogeneous [5]. Results from 23 pooled longitudinal studies with 45,734 participants found that both loneliness scores (19 studies) and prevalence (8 studies) increased relative to pre-pandemic times, with small effect sizes [23]. The evidence suggests that participants’ mental health may have been influenced by the pandemic, especially at the beginning of the pandemic. Further research could address the trajectory of mental health and seek interventions to help the public recover from poorer mental health in the post-pandemic period.

Various factors, e.g., differences in the lockdown duration, the countermeasures taken by different governments, and the intensity of the pandemic, may contribute to the heterogeneity of the results. The former meta-analysis reported a linear association of worsening depression and anxiety with increasing stringency in governmental measures [5]. This was supported by another multi-country analysis that recruited 432,642 responses from 15 countries, which found that more stringent COVID-19 policies were associated with more severe psychological distress [24]. Interestingly, another meta-analysis that enrolled 114 studies from 33 countries (*N* = 640,037) reported that depressive symptoms were lower in countries wherein governments implemented stringent policies promptly [25]. A study estimated the associations between lockdown stringency and duration with Google search for mental health and found that overall lockdown stringency, lockdown duration, the most stringent stay-at-home requirements, and policies recommending or requiring the cancellation of public events were associated with less depression and anxiety, but policies requiring school closures were associated with more severe depression [26]. More studies are warranted to balance the benefits and risks of countermeasures.

Our results showed that severe mental health symptoms and poor self-rated health were positively associated with each other, and pre-existing health problems increased the risk of poor health during the pandemic. In a study comprising more than 1.5 million community-dwelling American adults, depressive symptoms were negatively associated with health during the pandemic [14]. In another study, COVID anxiety was negatively associated with better health [15]. Former research within the NAKO also reported that having a self-reported psychiatric history increased the risk of PHQ-9 and GAD-7 symptoms [27]. Supporting our findings, research with 6,038 German participants reported that individuals with a history of depressive symptoms prior to the COVID-19 pandemic were at increased risk of experiencing an escalation of mental health problems due to the COVID-19 pandemic [28]. The increased risk of worsening mental health during the pandemic among individuals with pre-existing mental disorders may be due to an increased genetic and/or environmental vulnerability and the disruption of healthcare during the pandemic. These findings suggest the importance and need for early identification and intervention of mental problems.

Our findings support that having more sport-related physical activity during the pandemic was associated with improved self-rated health. The health benefits of physical activity have been well established. However, the pandemic and related containment measures restricted the activity of the population. In our population, only 19.5% of participants reported more sport-related physical activity, while 28.5% of participants had less physical activity during the pandemic. The former study reported that participants in NAKO engaged in fewer sports activities during the pandemic compared to baseline [29]. Consistent with our findings, researchers in a meta-analysis with a total sample size of 119,094 participants aged 4 to 93 years (57 studies from 14 countries worldwide), reported that most self-rated and all device-based physical activities showed a reduction during the pandemic [30]. The reductions in physical activity suggest that strategies motivating populations to be more active should be implemented, especially during public health emergencies. Randomized controlled trial has shown that in times of restricted physical activity during the pandemic, home-based exercise programs [31] may constitute alternatives to counteract physical inactivity and preserve/improve health. However, our analysis could not clarify the causal relationship between physical activity and health status; caution should be taken in results interpretation.

Meta-analyses of randomized trials showed that exercise was an effective treatment for depression [32]. In causal inference studies, research found the protective effect of objectively assessed but not self-reported physical activity on reduced depression [33]. In our study, we collected the self-reported changes in physical activity during the pandemic and observed that both increased and decreased physical activity increased mental health scores. Self-reported measures of activity can be influenced by mood states and cognitive biases that also affect mental health, making it difficult to ascertain whether observed associations are true or simply artifacts of a shared liability. For example, individuals vulnerable to mental health may perceive themselves as more inactive and disengaged than their peers or compensate by overreporting activity. We collected information on changes in physical activity and mental health data simultaneously, making it impossible to determine their temporal sequence.

Our results showed that the proportions of current smokers and heavy drinkers decreased during the pandemic compared with baseline. Consistent with our findings, the meta-analysis reported a relative reduction in the overall prevalence of smoking [2] and alcohol consumption during the pandemic [34]. However, two other systematic reviews showed an increase in alcohol consumption during the pandemic [3]. In our study, we found that 15.3% of former smokers at baseline reported they never smoked during the pandemic survey. We collected self-reported smoking data from the questionnaire. The previous systematic review reported the bias of self-reported smoking [35]. In our study, we cannot conclude which survey is reliable without cotinine measurement. In the association analysis, we evaluated changes in smoking between never smokers (never or past smokers) and current smokers to exclude potential confounding.

In the association analysis, we found that smoking was associated with worse self-rated and mental health, whereas heavy drinking was associated with more severe mental health symptoms but better self-rated health. A former prospective study revealed that an unfavorable lifestyle, most likely driven by BMI, smoking, physical activity, and sleep duration, was associated with the risk of depression and anxiety, but drinking did not affect mental health during the pandemic [36]. Another study found that higher levels of alcohol consumption during the first wave of the pandemic were associated with higher levels of depression and anxiety in the following year [37], another study reported bidirectional associations between them [38]. In contrast to our findings, earlier research reported that alcohol consumption was a stronger predictor of poor self-rated health [39]. We collected data on health perception and alcohol consumption simultaneously. Although our longitudinal design enhances reliability, potential reverse causality may also be introduced. “Sick quitter effect/bias” might be another explanation, as ex-drinkers usually disproportionally have poor health status [40]. A previous study reported that pandemic-related worries were associated with less alcohol consumption, which was mediated by loneliness [41]. So, social alcohol consumption, which alleviates worries during the pandemic, might also contribute to our findings.

In the multivariable regression, we identified participants’ characteristics associated with poor health status and worse health change. Our results showed that being older was associated with poor self-rated health but good mental health. Consistent with our findings, some studies found that being older increased the risk of poor/worsened self-rated health during the pandemic [11, 13, 14], but others reported the opposite association [9, 10, 15]. For mental health, current research supports that adolescents and young adults are likely to be disproportionately affected [22], which may be the result of unfavorable behavioral and social changes (for example, school closure periods) during a crucial development phase when social interactions outside the family context are pivotal. We found that being female was associated with higher risks of poor health and worse health change. Current evidence supports that female participants suffered worsening self-rated health [10] and mental health [22]. The evidence regarding self-rated health status appears to be contradictory; with some studies finding that being female increases the risk of poor health [14], others finding that it decreases the risk of poor health, and still others reporting no sex difference [9]. A former NAKO study found that occupational and financial difficulties were essential contributors to increased depressive symptoms and anxiety disorders during the first year of the COVID-19 pandemic [42]. In our data, employed participants had better self-rated health and depression symptoms but worse anxiety and stress scales. We also found that deteriorated financial status was positively associated with better self-rated health but more severe mental health symptoms. Factors like access to healthcare, including testing for infections that increase the risk of poor health, and therapeutic resources, as well as language, educational, and technological barriers to learning and engaging in preventive measures, may contribute to the socioeconomic disparity. However, it remains unclear whether the impact is greater for individuals with low socio-economic status, with contrasting meta-analyses suggesting that this group is protected [43] or at increased risk [22]. Our study reported that having had a coronavirus test increased the risk of poor health. A meta-analysis demonstrated that concerns about being infected were repeatedly reported as risk factors for poor mental health [22].

The main strength of our analysis is that the results are derived from a large, population-based cohort sampled from 18 study centers in Germany. The baseline information provides detailed characterizations of our participants before the outbreak of COVID-19, allowing comparison of health status between the baseline and the early phase of the pandemic, as well as identification of participants’ characteristics during the pandemic under the control of baseline characteristics. Despite this strength, our study has several limitations. First, we collected all information from a self-reported questionnaire, the results of which are highly dependent on personal perception. Reverse causation cannot be ruled out, as we collected all information at the same time in each survey. It is also impossible to distinguish the contributions of the pandemic, the countermeasures, or other factors to health status. Second, we recruited only a sample of the German population, so the results cannot be generalized to other populations. Different populations have specific characteristics, and different countries implement different measures during the pandemic. Furthermore, fewer than 60% of participants in the original cohort responded to the COVID surveys during our study period, potentially introducing selection bias into our results. Nevertheless, the relatively stable results from the sensitivity analysis with inverse probability weighting suggest that our findings might not be significantly influenced.

## Conclusions

In conclusion, the participants in our study reported an improvement in self-rated health but deteriorations in physical activity and mental health, especially for stress, from baseline to the early pandemic when corresponding countermeasures were induced. Our findings suggest that the public may benefit from strategies for early identification and intervention of participants with health problems, as well as from programs to promote increased physical activity during the pandemic. Future studies could address the long-term effects of the pandemic on public health and strategies to improve public health in the post-pandemic era.

## Supplementary Information

Supplementary Material 1.

## Data Availability

The datasets analyzed during the current study are not publicly available. Access to and use of NAKO data and biosamples can be obtained via an electronic application portal (https://transfer.nako.de). Analysis codes are available from the authors upon request.
