# Supplementary material for: Self-rated health and mental health before and during the early phase of the COVID-19 pandemic in Germany: the population-based German National Cohort (NAKO) study
Source: BMC Public Health. 2026 May 9;26:1512. doi: 10.1186/s12889-026-27633-5 (PMC13156848; doi:10.1186/s12889-026-27633-5)
Supplement: Supplementary file 1 — Supplementary Material 1. [file 12889_2026_27633_MOESM1_ESM.docx]

**Self-rated health and mental health before and during the early phase of the COVID-19 pandemic in Germany: the population-based German National Cohort (NAKO) study**

Supplementary

__________________________________________________________________________*Corresponding author:

*Yue Xi*

Institute of Epidemiology, Helmholtz Zentrum München - German Research Center for Environmental Health

Ingolstädter Landstr. 1, 85764 Neuherberg, Germany

E-Mail: [yue.xi@helmholtz-munich.de](mailto:yue.xi@helmholtz-munich.de)

**Methods**

**Assessment of mental health**

We used several modules from the German version of the Patient Health Questionnaire (PHQ) to assess mental health: depressive symptoms (PHQ-9), anxiety symptoms (GAD-7), and perceived psychosocial strains (PHQ-stress)[1, 2]. Summary scores for all three mental health scales were calculated according to the PHQ manual. The respective ranges of the scores are 0 to 27 points for PHQ-9, 0 to 21 points for GAD-7, and 0 to 20 points for PHQ-stress. The PHQ-9, GAD-7, and PHQ-stress have been well-validated as efficient mental health diagnostic tools[3-5]. We used a cut-off score of 10 to divide individuals into two groups, based on which we created four change categories: those who maintained a score <10, those who increased from <10 to ≥ 10, those who decreased from ≥10 to <10, and those who maintained a score ≥10 [4, 6].

**Examination of factors potentially associated with self-rated health and mental health**

Education status at baseline was divided into university and no university. Employment status was divided into employed and unemployed. Cohabitation was grouped into living with others and living alone. Change in household financial status (no change, deteriorated, and improved) in the COVID-NAKO questionnaire. Cigarette smoking status was grouped into never, former, and current. Alcohol consumption frequency at baseline examination and in the COVID-NAKO questionnaire was categorized into never, ≤ 1 time /month, 2 to 4 times /month, 2 to 4 times /week, 5-6 times /week, and every day. Due to the small sample size of never and daily drinkers, we regrouped participants into the following four groups for association analysis: never or ≤ 1 time /month, 2 to 4 times /month, 2 to 4 times /week, and ≥ 5 times /week. Changes in physical activity (household, leisure-time, and sports-related) and sitting behavior were reclassified as less, same, or more. To measure the lifestyle changes from baseline to the COVID-NAKO survey, we divided participants into four groups with respect to smoking (no smoking, start smoking, quit smoking, continue smoking) and into three groups with respect to alcohol consumption (less, same, or more). Life satisfaction at baseline examination ranged from 0 to 10 points; a higher score indicates higher satisfaction.

**Statistical analysis**

In the descriptive analysis, categorical variables were presented as frequencies and percentages, continuous variables were presented as mean and standard deviation for variables with normal distributions, and median and interquartile range for others.

In the multinomial logistic regression models, we chose “Excellent” and “Same” as reference groups for health status and health change, respectively. We first included lifestyle and mental health status during the pandemic, and then replaced status with their changes from baseline to COVID-NAKO questionnaire.

In the linear and quantile regression, to examine potentially associated factors of mental health scales and their changes. We also fitted two models to examine the associations of lifestyle factors and their changes for each of our outcomes. To eliminate bias from baseline health status, we adjusted each health outcome for baseline health status and time since baseline examination.

**Reference**

1. Spitzer, R.L., K. Kroenke, and J.B. Williams, *Validation and utility of a self-report version of PRIME-MD: the PHQ primary care study. Primary Care Evaluation of Mental Disorders. Patient Health Questionnaire.* JAMA, 1999. **282**(18): p. 1737-1744.

2. Löwe, B., et al., *Detecting panic disorder in medical and psychosomatic outpatients: comparative validation of the Hospital Anxiety and Depression Scale, the Patient Health Questionnaire, a screening question, and physicians' diagnosis.* Journal of Psychosomatic Research, 2003. **55**(6): p. 515-519.

3. Löwe, B., et al., *Monitoring depression treatment outcomes with the patient health questionnaire-9.* Medical Care, 2004. **42**(12): p. 1194-1201.

4. Spitzer, R.L., et al., *A brief measure for assessing generalized anxiety disorder: the GAD-7.* Archives of Internal Medicine, 2006. **166**(10): p. 1092-1097.

5. Petrowski, K., et al., *Psychometric evaluation of the patient health questionnaire stress scale.* Journal of Affective Disorders, 2024. **357**: p. 37-41.

6. Kroenke, K., R.L. Spitzer, and J.B. Williams, *The PHQ-9: validity of a brief depression severity measure.* Journal of General Internal Medicine, 2001. **16**(9): p. 606-613.

**Results**

Fig. S1. Flow diagram for the study.


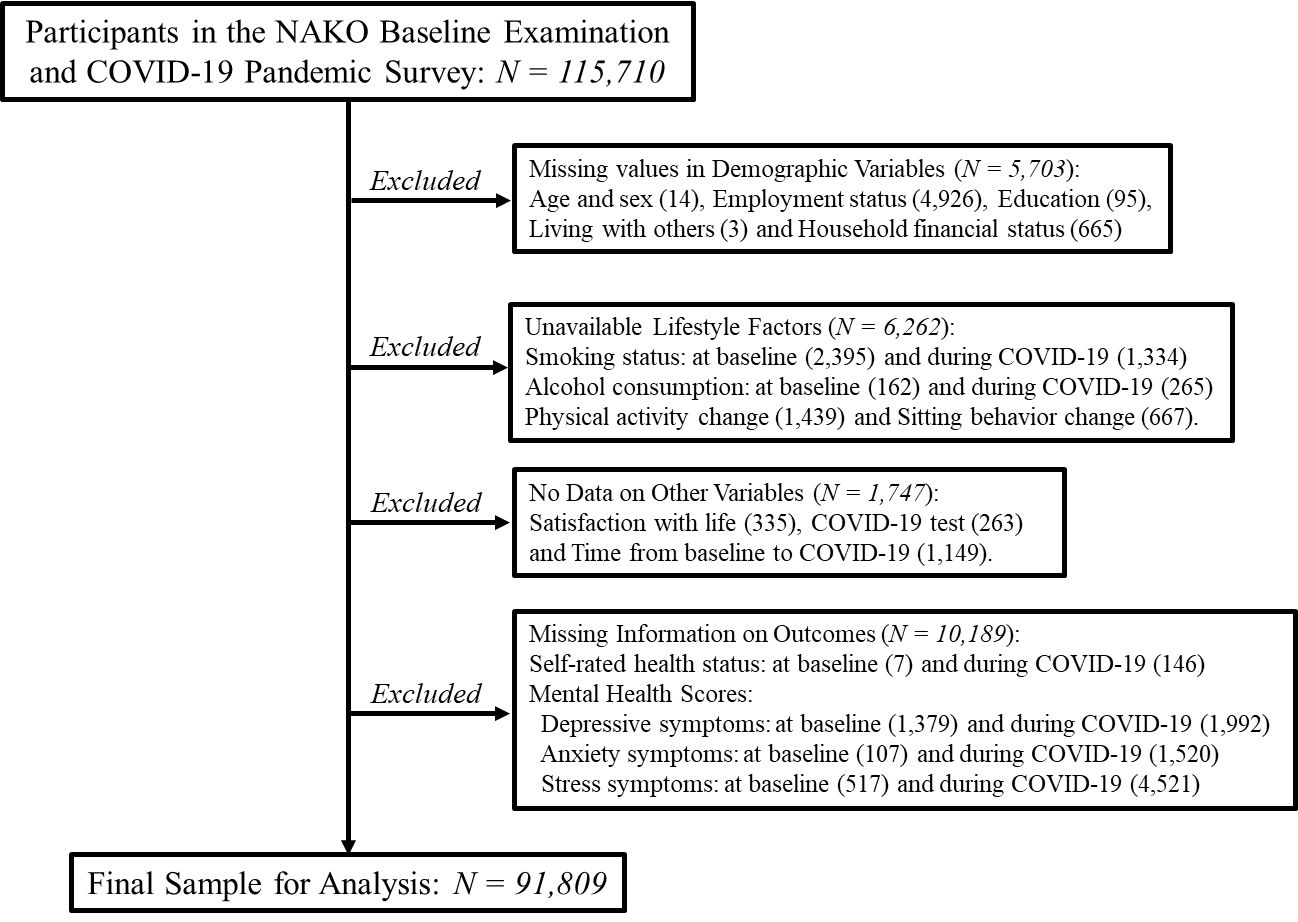


TABLE S1: Factors associated with self-rated health status during early COVID-19.

| Variables | Very Good | Good | Less Good to Bad |
| --- | --- | --- | --- |
| Age at baseline (years) | 1.02(1.02,1.02)* | 1.05(1.04,1.05)* | 1.07(1.06,1.07)* |
| Gender: Male | 0.78(0.74,0.81)* | 0.69(0.65,0.73)* | 0.75(0.70,0.82)* |
| Education at baseline: University | 1.04(0.99,1.09) | 0.88(0.83,0.93)* | 0.83(0.76,0.90)* |
| Employment status: Employed | 1.08(1.01,1.15) | 0.99(0.92,1.06) | 0.79(0.71,0.87)* |
| Cohabitation: Living with others | 1.15(1.07,1.23)* | 1.19(1.11,1.28)* | 1.05(0.95,1.16) |
| Financial status: Deteriorated | 0.94(0.89,1.00) | 0.89(0.83,0.95)* | 0.85(0.77,0.94)* |
| Financial status: Improved | 1.00(0.89,1.12) | 0.89(0.78,1.02) | 0.92(0.73,1.16) |
| Life satisfaction at baseline | 0.89(0.87,0.91)* | 0.81(0.79,0.82)* | 0.79(0.77,0.80)* |
| Coronavirus test | 1.02(0.91,1.15) | 1.40(1.23,1.58)* | 2.63(2.23,3.11)* |
| Household physical activity: Less | 1.01(0.87,1.17) | 1.31(1.12,1.53)* | 1.79(1.49,2.16)* |
| Household physical activity: More | 0.97(0.92,1.02) | 0.94(0.89,1.00) | 0.89(0.81,0.97) |
| Sport-related physical activity: Less | 1.14(1.08,1.21)* | 1.20(1.13,1.28)* | 1.27(1.17,1.39)* |
| Sport-related physical activity: More | 0.80(0.76,0.85)* | 0.58(0.54,0.62)* | 0.46(0.41,0.53)* |
| Sitting behavior: Less | 0.97(0.89,1.07) | 0.95(0.86,1.05) | 1.13(0.98,1.31) |
| Sitting behavior: More | 1.12(1.06,1.19)* | 1.24(1.17,1.32)* | 1.41(1.29,1.54)* |
| Smoking status: Past smoker | 1.03(0.97,1.09) | 1.11(1.05,1.18)* | 1.21(1.11,1.32)* |
| Smoking status: Current smoker | 1.07(0.99,1.16) | 1.25(1.15,1.36)* | 1.38(1.23,1.55)* |
| Alcohol consumption: 2 to 4 times/month | 1.10(1.03,1.17)* | 1.05(0.98,1.13) | 0.80(0.72,0.89)* |
| Alcohol consumption: 2 to 4 times/week | 1.09(1.02,1.16) | 0.94(0.87,1.01) | 0.62(0.56,0.69)* |
| Alcohol consumption: ≥ 5 times/week | 1.11(1.03,1.21) | 0.98(0.90,1.07) | 0.63(0.56,0.72)* |
| Depression score: ≥10 | 1.23(1.07,1.43)* | 2.38(2.05,2.77)* | 6.27(5.29,7.44)* |
| Anxiety score: ≥10 | 1.34(1.12,1.61)* | 1.64(1.36,1.98)* | 2.93(2.38,3.61)* |
| Stress score: ≥10 | 1.33(1.18,1.51)* | 2.32(2.05,2.64)* | 3.50(3.02,4.06)* |
| Smoking change: Yes to No^b^ | 0.89(0.80,0.99) | 0.98(0.87,1.11) | 1.20(1.00,1.43) |
| Smoking change: No to Yes^b^ | 1.09(0.92,1.30) | 1.10(0.91,1.34) | 1.17(0.87,1.56) |
| Smoking change: Remained Yes^b^ | 1.07(0.98,1.16) | 1.23(1.12,1.34)* | 1.30(1.15,1.46)* |
| Alcohol consumption change: Less^b^ | 0.97(0.91,1.02) | 1.03(0.97,1.10) | 1.27(1.16,1.39)* |
| Alcohol consumption change: More^b^ | 1.00(0.94,1.06) | 0.99(0.93,1.06) | 0.81(0.73,0.89)* |
| Depression change: Decreased from ≥10 to <10^b^ | 0.82(0.67,0.99) | 0.82(0.67,1.00) | 1.00(0.79,1.27) |
| Depression change: Increased from <10 to ≥10^b^ | 1.22(1.04,1.42) | 2.45(2.09,2.87)* | 6.98(5.80,8.40)* |
| Depression change: Remained ≥10^b^ | 1.33(0.92,1.93) | 2.22(1.53,3.22)* | 5.76(3.89,8.53)* |
| Anxiety change: Decreased from ≥10 to <10^b^ | 0.80(0.64,1.00) | 0.69(0.55,0.87)* | 0.63(0.48,0.83)* |
| Anxiety change: Increased from <10 to ≥10^b^ | 1.39(1.14,1.68)* | 1.69(1.38,2.07)* | 3.07(2.45,3.84)* |
| Anxiety change: Remained ≥10^b^ | 1.10(0.69,1.73) | 1.31(0.82,2.07) | 2.16(1.33,3.50)* |
| Stress change: Decreased from ≥10 to <10^b^ | 0.78(0.60,1.02) | 0.92(0.71,1.20) | 1.05(0.78,1.41) |
| Stress change: Increased from <10 to ≥10^b^ | 1.38(1.21,1.57)* | 2.41(2.11,2.75)* | 3.82(3.26,4.47)* |
| Stress change: Remained ≥10^b^ | 1.00(0.68,1.46) | 1.82(1.24,2.66)* | 2.58(1.73,3.85)* |

Note: The table presents the Relative Risk Ratio (RRR) and 95% confidence interval (CI) from the multinomial logistic regression model, using “Excellent” as the reference group.

The model included smoking, alcohol, and mental health scales during COVID-19 and was adjusted with health status at baseline and time from baseline to the COVID-NAKO survey.

^b^The model included variables’ changes from baseline to COVID-NAKO survey. *Statistically significant after Bonferroni correction.

TABLE S2. Factors associated with stress score during early COVID-19.

| Variables | Linear | Q10 | Q25 | Q50 | Q75 | Q90 |
| --- | --- | --- | --- | --- | --- | --- |
| Age at baseline(years) | -0.02(-0.02,-0.02)* | -0.01(-0.01,-0.01)* | -0.01(-0.02,-0.01)* | -0.02(-0.02,-0.02)* | -0.03(-0.03,-0.02)* | -0.03(-0.04,-0.03)* |
| Gender: Male | -0.40(-0.44,-0.37)* | -0.19(-0.22,-0.16)* | -0.29(-0.32,-0.25)* | -0.41(-0.45,-0.37)* | -0.50(-0.55,-0.44)* | -0.52(-0.60,-0.44)* |
| Education at baseline: University | -0.03(-0.06,0.01) | 0.10(0.08,0.13)* | 0.11(0.07,0.14)* | 0.02(-0.02,0.07) | -0.11(-0.17,-0.05)* | -0.30(-0.38,-0.22)* |
| Employment status: Employed | 0.49(0.44,0.53)* | 0.14(0.11,0.17)* | 0.26(0.22,0.30)* | 0.40(0.35,0.45)* | 0.64(0.57,0.70)* | 0.85(0.75,0.95)* |
| Cohabitation: Living with others | 0.29(0.24,0.33)* | 0.11(0.07,0.14)* | 0.19(0.14,0.23)* | 0.30(0.24,0.35)* | 0.34(0.26,0.41)* | 0.37(0.27,0.47)* |
| Financial status: Deteriorated | 0.99(0.95,1.03)* | 0.51(0.46,0.56)* | 0.75(0.70,0.80)* | 0.99(0.93,1.05)* | 1.21(1.13,1.29)* | 1.38(1.27,1.48)* |
| Financial status: Improved | -0.13(-0.23,-0.04)* | 0.05(-0.03,0.13) | 0.02(-0.05,0.10) | -0.08(-0.18,0.02) | -0.24(-0.37,-0.11)* | -0.33(-0.53,-0.13)* |
| Self-rated health status | -0.01(-0.02,0.00) | -0.00(-0.01,0.01) | -0.01(-0.02,0.01) | -0.02(-0.03,-0.00) | -0.02(-0.04,-0.00) | -0.04(-0.06,-0.02)* |
| Life satisfaction at baseline | 0.76(0.73,0.78)* | 0.42(0.40,0.44)* | 0.60(0.57,0.62)* | 0.75(0.72,0.78)* | 0.90(0.86,0.94)* | 0.98(0.93,1.04)* |
| Coronavirus test | 0.36(0.28,0.44)* | 0.26(0.17,0.35)* | 0.38(0.29,0.47)* | 0.34(0.25,0.44)* | 0.40(0.28,0.52)* | 0.47(0.32,0.63)* |
| Household physical activity: Less | 0.82(0.73,0.90)* | 0.57(0.48,0.66)* | 0.77(0.67,0.87)* | 0.82(0.71,0.93)* | 0.98(0.83,1.13)* | 1.01(0.82,1.21)* |
| Household physical activity: More | 0.34(0.30,0.38)* | 0.24(0.21,0.27)* | 0.29(0.25,0.34)* | 0.35(0.30,0.40)* | 0.40(0.34,0.46)* | 0.40(0.31,0.48)* |
| Sport-related physical activity: Less | 0.53(0.49,0.57)* | 0.31(0.28,0.34)* | 0.39(0.35,0.43)* | 0.53(0.48,0.57)* | 0.61(0.55,0.67)* | 0.67(0.59,0.76)* |
| Sport-related physical activity: More | 0.10(0.05,0.15)* | 0.07(0.03,0.10)* | 0.09(0.04,0.13)* | 0.12(0.06,0.17)* | 0.11(0.03,0.19)* | 0.15(0.04,0.25)* |
| Sitting behavior: Less | 0.36(0.29,0.42)* | 0.06(-0.01,0.13) | 0.20(0.12,0.27)* | 0.36(0.27,0.44)* | 0.49(0.38,0.60)* | 0.59(0.43,0.75)* |
| Sitting behavior: More | 0.42(0.38,0.46)* | 0.33(0.29,0.37)* | 0.44(0.40,0.48)* | 0.46(0.41,0.51)* | 0.44(0.38,0.51)* | 0.43(0.35,0.52)* |
| Smoking status: Past smoker | 0.10(0.06,0.14)* | 0.04(0.01,0.07)* | 0.06(0.02,0.10)* | 0.14(0.09,0.19)* | 0.15(0.09,0.21)* | 0.16(0.07,0.24)* |
| Smoking status: Current smoker | 0.00(-0.05,0.05) | -0.06(-0.10,-0.02)* | -0.05(-0.11,0.01) | 0.02(-0.05,0.08) | 0.05(-0.03,0.14) | 0.04(-0.07,0.16) |
| Alcohol consumption: 2 to 4 times/month | 0.02(-0.02,0.07) | 0.03(-0.01,0.07) | 0.02(-0.03,0.07) | 0.05(-0.00,0.11) | -0.01(-0.09,0.07) | -0.05(-0.16,0.06) |
| Alcohol consumption: 2 to 4 times/week | 0.04(-0.01,0.08) | 0.04(0.01,0.08) | 0.07(0.03,0.12)* | 0.08(0.02,0.13)* | 0.00(-0.07,0.08) | -0.09(-0.19,0.02) |
| Alcohol consumption: ≥ 5 times/week | 0.12(0.07,0.18)* | 0.10(0.05,0.14)* | 0.12(0.07,0.17)* | 0.14(0.08,0.20)* | 0.08(-0.00,0.17) | -0.02(-0.14,0.10) |
| Depression score: ≥10 | 1.94(1.87,2.02)* | 1.74(1.65,1.84)* | 1.93(1.82,2.03)* | 2.05(1.94,2.16)* | 2.12(1.98,2.25)* | 2.02(1.88,2.17)* |
| Anxiety score: ≥10 | 1.96(1.87,2.05)* | 2.03(1.87,2.19)* | 2.05(1.93,2.16)* | 2.00(1.86,2.13)* | 2.00(1.83,2.16)* | 2.08(1.92,2.25)* |
| Smoking change: Yes to No^b^ | 0.02(-0.06,0.11) | -0.01(-0.08,0.06) | 0.02(-0.08,0.12) | 0.09(0.00,0.19) | 0.05(-0.06,0.16) | 0.04(-0.13,0.21) |
| Smoking change: No to Yes^b^ | 0.07(-0.06,0.20) | -0.06(-0.22,0.09) | -0.06(-0.21,0.10) | 0.08(-0.12,0.28) | 0.25(-0.04,0.53) | 0.35(0.07,0.62) |
| Smoking change: Remained Yes^b^ | -0.05(-0.10,0.00) | -0.08(-0.11,-0.04)* | -0.07(-0.14,-0.01) | -0.03(-0.09,0.03) | -0.05(-0.14,0.04) | -0.06(-0.19,0.06) |
| Alcohol consumption change: Less^b^ | -0.01(-0.05,0.04) | -0.04(-0.06,-0.01) | -0.04(-0.08,-0.00) | -0.01(-0.06,0.04) | 0.04(-0.02,0.11) | 0.04(-0.05,0.14) |
| Alcohol consumption change: More^b^ | 0.23(0.19,0.28)* | 0.09(0.06,0.12)* | 0.15(0.10,0.20)* | 0.24(0.19,0.30)* | 0.30(0.23,0.37)* | 0.32(0.22,0.42)* |

Note: The table presents the absolute changes in stress scale and 95% CI from the linear/quantile regression model.

The model included smoking, alcohol, and mental health scales during the COVID-19 and was adjusted with stress score at baseline and time from baseline to the COVID-NAKO survey

^b^The model included variables’ changes from baseline to the COVID-NAKO survey. *Statistically significant after Bonferroni correction.

TABLE S3. Factors associated with anxiety score during early COVID-19.

| Variables | Linear | Q10 | Q25 | Q50 | Q75 | Q90 |
| --- | --- | --- | --- | --- | --- | --- |
| Age at baseline(years) | -0.02(-0.02,-0.02)* | -0.00(-0.00, 0.00) | -0.02(-0.02,-0.01)* | -0.02(-0.02,-0.02)* | -0.03(-0.03,-0.03)* | -0.03(-0.03,-0.03)* |
| Gender: Male | -0.50(-0.53,-0.47)* | -0.00(-0.02,0.02) | -0.27(-0.29,-0.25)* | -0.50(-0.53,-0.47)* | -0.68(-0.72,-0.63)* | -0.75(-0.82,-0.68)* |
| Education at baseline: University | 0.24(0.21,0.28)* | 0.00(-0.02,0.02) | 0.17(0.15,0.19)* | 0.26(0.22,0.29)* | 0.30(0.25,0.34)* | 0.26(0.19,0.32)* |
| Employment status: Employed | 0.12(0.08,0.16)* | 0.00(-0.02,0.02) | 0.03(0.01,0.06)* | 0.15(0.11,0.19)* | 0.17(0.12,0.23)* | 0.15(0.06,0.24)* |
| Cohabitation: Living with others | 0.15(0.11,0.20)* | 0.00(-0.02,0.02) | 0.11(0.09,0.14)* | 0.19(0.14,0.23)* | 0.20(0.14,0.27)* | 0.14(0.04,0.23)* |
| Financial status: Deteriorated | 0.48(0.44,0.52)* | 0.00(-0.02,0.02) | 0.29(0.25,0.33)* | 0.48(0.44,0.53)* | 0.59(0.53,0.65)* | 0.76(0.68,0.85)* |
| Financial status: Improved | -0.10(-0.18,-0.01) | -0.00(-0.05,0.05) | -0.05(-0.09,-0.01) | -0.07(-0.15,0.01) | -0.11(-0.22,0.01) | -0.09(-0.26,0.08) |
| Self-rated health status | 0.50(0.47,0.52)* | 0.00(-0.01,0.01) | 0.28(0.27,0.29)* | 0.48(0.46,0.50)* | 0.61(0.57,0.64)* | 0.65(0.61,0.70)* |
| Life satisfaction at baseline | 0.01(0.00,0.02) | -0.00(-0.01,0.01) | -0.01(-0.01,-0.00) | 0.00(-0.01,0.01) | -0.00(-0.02,0.01) | 0.00(-0.02,0.02) |
| Coronavirus test | 0.21(0.13,0.28)* | 0.00(-0.04,0.04) | 0.13(0.05,0.21)* | 0.14(0.06,0.23)* | 0.25(0.13,0.36)* | 0.35(0.18,0.52)* |
| Household physical activity: Less | 0.54(0.46,0.62)* | 0.00(-0.04,0.04) | 0.39(0.30,0.49)* | 0.64(0.53,0.75)* | 0.80(0.67,0.93)* | 0.80(0.64,0.95)* |
| Household physical activity: More | 0.26(0.22,0.29)* | 0.00(-0.02,0.02) | 0.19(0.16,0.22)* | 0.29(0.25,0.33)* | 0.32(0.27,0.37)* | 0.35(0.27,0.42)* |
| Sport-related physical activity: Less | 0.21(0.18,0.24)* | 0.00(-0.02,0.02) | 0.14(0.12,0.16)* | 0.23(0.19,0.27)* | 0.24(0.19,0.29)* | 0.22(0.14,0.29)* |
| Sport-related physical activity: More | 0.15(0.10,0.19)* | 0.00(-0.02,0.02) | 0.08(0.05,0.11)* | 0.17(0.13,0.22)* | 0.18(0.12,0.24)* | 0.22(0.13,0.31)* |
| Sitting behavior: Less | 0.27(0.21,0.34)* | -0.00(-0.02,0.02) | 0.08(0.03,0.12)* | 0.23(0.16,0.31)* | 0.35(0.25,0.45)* | 0.47(0.34,0.61)* |
| Sitting behavior: More | 0.23(0.19,0.26)* | 0.00(-0.02,0.02) | 0.18(0.15,0.21)* | 0.26(0.22,0.30)* | 0.27(0.22,0.32)* | 0.34(0.26,0.41)* |
| Smoking status: Past smoker | 0.03(0.00,0.07) | 0.00(-0.02,0.02) | 0.04(0.03,0.06)* | 0.06(0.03,0.10)* | 0.06(0.01,0.11) | 0.02(-0.05,0.09) |
| Smoking status: Current smoker | -0.05(-0.10,0.00) | 0.00(-0.02,0.02) | -0.03(-0.06,-0.00) | -0.02(-0.07,0.03) | -0.02(-0.09,0.05) | -0.01(-0.11,0.10) |
| Alcohol consumption: 2 to 4 times/month | 0.07(0.03,0.11)* | 0.00(-0.02,0.02) | 0.08(0.06,0.11)* | 0.10(0.05,0.14)* | 0.05(-0.01,0.11) | 0.03(-0.06,0.13) |
| Alcohol consumption: 2 to 4 times/week | 0.18(0.14,0.23)* | 0.00(-0.02,0.02) | 0.15(0.12,0.18)* | 0.21(0.17,0.26)* | 0.19(0.13,0.25)* | 0.13(0.04,0.21)* |
| Alcohol consumption: ≥ 5 times/week | 0.29(0.24,0.34)* | 0.00(-0.02,0.02) | 0.16(0.14,0.19)* | 0.25(0.20,0.31)* | 0.33(0.26,0.40)* | 0.30(0.19,0.42)* |
| Depression score: ≥10 | 4.43(4.37,4.49)* | 4.00(3.94,4.06)* | 3.61(3.52,3.69)* | 4.06(3.94,4.17)* | 5.08(4.94,5.23)* | 6.11(5.91,6.30)* |
| Stress score: ≥10 | 2.00(1.94,2.05)* | 2.00(1.96,2.04)* | 1.94(1.88,2.00)* | 2.04(1.96,2.11)* | 2.12(2.02,2.22)* | 2.28(2.14,2.42)* |
| Smoking change: Yes to No^b^ | 0.05(-0.03,0.12) | -0.00(-0.04,0.04) | 0.00(-0.05,0.05) | 0.05(-0.02,0.13) | 0.13(0.01,0.25) | 0.17(0.03,0.31) |
| Smoking change: No to Yes^b^ | 0.02(-0.09,0.14) | 0.00(-0.06,0.06) | 0.07(-0.05,0.18) | 0.11(-0.00,0.23) | 0.09(-0.09,0.28) | 0.04(-0.23,0.30) |
| Smoking change: Remained Yes^b^ | -0.05(-0.10,-0.01) | -0.00(-0.02,0.02) | -0.06(-0.09,-0.03)* | -0.05(-0.10,-0.00) | -0.03(-0.10,0.04) | -0.01(-0.11,0.10) |
| Alcohol consumption change: Less^b^ | -0.04(-0.08,0.00) | -0.00(-0.01,0.01) | -0.04(-0.06,-0.03)* | -0.05(-0.09,-0.01) | -0.01(-0.07,0.04) | 0.03(-0.05,0.10) |
| Alcohol consumption change: More^b^ | 0.18(0.14,0.22)* | -0.00(-0.02,0.02) | 0.09(0.06,0.12)* | 0.12(0.08,0.17)* | 0.22(0.16,0.27)* | 0.29(0.21,0.38)* |

Note: The table presents the absolute changes in anxiety scale and 95% CI from the linear/quantile regression model.

The model included smoking, alcohol, and mental health scales during COVID-19 and was adjusted with anxiety score at baseline and time from baseline to the COVID-NAKO survey.

^b^The model included variables’ changes from baseline to the COVID-NAKO survey. *Statistically significant after Bonferroni correction.

TABLE S4: Factors associated with depression score change from baseline examination to early COVID-19.

| Variables | Linear | Q10 | Q25 | Q50 | Q75 | Q90 |
| --- | --- | --- | --- | --- | --- | --- |
| Age at baseline(years) | -0.04(-0.05,-0.04)* | -0.02(-0.02,-0.01)* | -0.03(-0.03,-0.03)* | -0.04(-0.04,-0.04)* | -0.05(-0.06,-0.05)* | -0.06(-0.07,-0.06)* |
| Gender: Male | -0.33(-0.36,-0.29)* | -0.12(-0.13,-0.10)* | -0.24(-0.27,-0.21)* | -0.38(-0.41,-0.34)* | -0.44(-0.49,-0.39)* | -0.48(-0.56,-0.41)* |
| Education at baseline: University | 0.16(0.12,0.19) | 0.09(0.08,0.10)* | 0.15(0.11,0.18)* | 0.16(0.12,0.20)* | 0.17(0.12,0.23)* | 0.16(0.08,0.23)* |
| Employment status: Employed | -0.10(-0.15,-0.06)* | -0.03(-0.05,-0.01)* | -0.01(-0.05,0.03) | -0.01(-0.06,0.04) | -0.08(-0.14,-0.01) | -0.16(-0.26,-0.07)* |
| Cohabitation: Living with others | -0.44(-0.49,-0.40)* | -0.11(-0.14,-0.09)* | -0.22(-0.26,-0.17)* | -0.30(-0.35,-0.25)* | -0.45(-0.53,-0.37)* | -0.73(-0.83,-0.62)* |
| Financial status: Deteriorated | 0.51(0.47,0.56)* | 0.16(0.13,0.18)* | 0.30(0.25,0.34)* | 0.41(0.36,0.47)* | 0.68(0.61,0.76)* | 0.86(0.76,0.96)* |
| Financial status: Improved | -0.10(-0.20,-0.01)* | 0.02(-0.03,0.06) | -0.09(-0.18,0.01) | -0.12(-0.22,-0.03) | -0.08(-0.21,0.06) | -0.06(-0.23,0.11) |
| Self-rated health status | 0.84(0.81,0.87)* | 0.28(0.26,0.29)* | 0.54(0.52,0.56)* | 0.76(0.74,0.79)* | 0.95(0.92,0.99)* | 1.11(1.05,1.16)* |
| Life satisfaction at baseline | -0.04(-0.05,-0.03)* | -0.01(-0.02,-0.01)* | -0.03(-0.04,-0.02)* | -0.04(-0.05,-0.03)* | -0.06(-0.08,-0.04)* | -0.06(-0.08,-0.04)* |
| Coronavirus test | -0.01(-0.09,0.07)* | 0.04(-0.00,0.09) | 0.03(-0.05,0.11) | 0.01(-0.07,0.09) | 0.02(-0.10,0.13) | -0.01(-0.18,0.16) |
| Household physical activity: Less | 1.49(1.40,1.57) | 0.87(0.69,1.04)* | 1.24(1.13,1.35)* | 1.50(1.39,1.62)* | 1.78(1.64,1.93)* | 2.01(1.72,2.30)* |
| Household physical activity: More | 0.23(0.19,0.27)* | 0.13(0.11,0.15)* | 0.21(0.17,0.25)* | 0.25(0.21,0.29)* | 0.32(0.26,0.37)* | 0.28(0.20,0.36)* |
| Sport-related physical activity: Less | 0.39(0.35,0.43)* | 0.16(0.15,0.17)* | 0.27(0.24,0.31)* | 0.36(0.31,0.40)* | 0.45(0.40,0.51)* | 0.50(0.42,0.59)* |
| Sport-related physical activity: More | 0.12(0.07,0.17)* | 0.07(0.05,0.08)* | 0.09(0.05,0.13)* | 0.14(0.09,0.19)* | 0.17(0.10,0.24)* | 0.17(0.06,0.27)* |
| Sitting behavior: Less | 0.23(0.16,0.30)* | -0.00(-0.03,0.02) | 0.09(0.03,0.15)* | 0.20(0.12,0.29)* | 0.34(0.24,0.45)* | 0.38(0.24,0.53)* |
| Sitting behavior: More | 0.49(0.45,0.53)* | 0.23(0.20,0.25)* | 0.38(0.35,0.42)* | 0.49(0.44,0.53)* | 0.57(0.51,0.63)* | 0.64(0.56,0.73)* |
| Smoking status: Past smoker | 0.11(0.07,0.14)* | 0.05(0.04,0.06)* | 0.07(0.03,0.10)* | 0.10(0.06,0.14)* | 0.15(0.10,0.21)* | 0.18(0.10,0.26)* |
| Smoking status: Current smoker | 0.11(0.06,0.17)* | 0.03(0.01,0.05)* | 0.01(-0.04,0.06) | 0.08(0.02,0.15) | 0.21(0.13,0.29)* | 0.20(0.08,0.32)* |
| Alcohol consumption: 2 to 4 times/month | 0.00(-0.04,0.05)* | 0.07(0.05,0.09)* | 0.08(0.04,0.12)* | 0.05(-0.00,0.10) | -0.03(-0.10,0.04) | -0.16(-0.27,-0.06)* |
| Alcohol consumption: 2 to 4 times/week | 0.10(0.05,0.14) | 0.10(0.09,0.12)* | 0.14(0.09,0.18)* | 0.16(0.11,0.21)* | 0.08(0.01,0.15) | -0.07(-0.18,0.03) |
| Alcohol consumption: ≥ 5 times/week | 0.24(0.18,0.29)* | 0.13(0.11,0.14)* | 0.17(0.12,0.22)* | 0.21(0.15,0.27)* | 0.23(0.15,0.31)* | 0.23(0.11,0.35)* |
| Anxiety score: ≥10 | 5.80(5.72,5.88)* | 4.81(4.69,4.94)* | 5.03(4.89,5.16)* | 5.62(5.47,5.77)* | 6.52(6.35,6.69)* | 7.20(6.96,7.45)* |
| Stress score: ≥10 | 2.09(2.03,2.15)* | 1.65(1.59,1.71)* | 1.90(1.81,1.98)* | 2.14(2.06,2.23)* | 2.32(2.20,2.43)* | 2.50(2.32,2.68)* |
| Smoking change: Yes to No^b^ | 0.11(0.02,0.19) | 0.00(-0.04,0.05) | 0.06(-0.02,0.13) | 0.18(0.08,0.28)* | 0.14(0.02,0.26) | 0.22(0.01,0.42) |
| Smoking change: No to Yes^b^ | 0.20(0.07,0.32)* | 0.05(0.01,0.08)* | 0.03(-0.09,0.15) | 0.13(-0.05,0.30) | 0.26(0.08,0.44)* | 0.23(0.02,0.44) |
| Smoking change: Remained Yes^b^ | 0.08(0.02,0.13)* | 0.01(-0.02,0.03) | -0.00(-0.05,0.05) | 0.05(-0.02,0.11) | 0.17(0.08,0.25)* | 0.15(0.04,0.26) |
| Alcohol consumption change: Less^b^ | -0.05(-0.10,-0.01) | -0.04(-0.06,-0.03)* | -0.08(-0.11,-0.04)* | -0.05(-0.09,-0.01) | -0.01(-0.07,0.05) | -0.00(-0.09,0.08) |
| Alcohol consumption change: More^b^ | 0.22(0.17,0.26)* | 0.06(0.04,0.08)* | 0.10(0.06,0.14)* | 0.16(0.11,0.20)* | 0.24(0.18,0.31)* | 0.35(0.26,0.44)* |

Note: The table presents the absolute changes in depression change and 95% CI from the linear/quantile regression model.

The model included smoking, alcohol, and mental health scales during COVID-19 and was adjusted with depression score at baseline and time from baseline to the COVID-NAKO survey.

^b^The model included variables’ changes from baseline to COVID-NAKO survey. *Statistically significant after Bonferroni correction.

TABLE S5: Factors associated with anxiety score change from baseline examination to early COVID-19.

| Variables | Linear | Q10 | Q25 | Q50 | Q75 | Q90 |
| --- | --- | --- | --- | --- | --- | --- |
| Age at baseline(years) | -0.02(-0.02,-0.02)* | -0.00(-0.00,0.00) | -0.02(-0.02,-0.01)* | -0.02(-0.02,-0.02)* | -0.03(-0.03,-0.03)* | -0.03(-0.03,-0.03)* |
| Gender: Male | -0.50(-0.53,-0.47)* | -0.00(-0.02,0.02) | -0.27(-0.29,-0.25)* | -0.50(-0.53,-0.47)* | -0.68(-0.72,-0.63)* | -0.75(-0.82,-0.68)* |
| Education at baseline: University | 0.24(0.21,0.28)* | 0.00(-0.02,0.02) | 0.17(0.15,0.19)* | 0.26(0.22,0.29)* | 0.30(0.25,0.34)* | 0.26(0.19,0.32)* |
| Employment status: Employed | 0.12(0.08,0.16)* | 0.00(-0.02,0.02) | 0.03(0.01,0.06)* | 0.15(0.11,0.19)* | 0.17(0.12,0.23)* | 0.15(0.06,0.24)* |
| Cohabitation: Living with others | 0.15(0.11,0.20)* | 0.00(-0.02,0.02) | 0.11(0.09,0.14)* | 0.19(0.14,0.23)* | 0.20(0.14,0.27)* | 0.14(0.04,0.23)* |
| Financial status: Deteriorated | 0.48(0.44,0.52)* | -0.00(-0.02,0.02) | 0.29(0.25,0.33)* | 0.48(0.44,0.53)* | 0.59(0.53,0.65)* | 0.76(0.68,0.85)* |
| Financial status: Improved | -0.10(-0.18,-0.01) | -0.00(-0.05,0.05) | -0.05(-0.09,-0.01) | -0.07(-0.15,0.01) | -0.11(-0.22,0.01) | -0.09(-0.26,0.08) |
| Self-rated health status | 0.50(0.47,0.52)* | 0.00(-0.01,0.01) | 0.28(0.27,0.29)* | 0.48(0.46,0.50)* | 0.61(0.57,0.64)* | 0.65(0.61,0.70)* |
| Life satisfaction at baseline | 0.01(0.00,0.02) | -0.00(-0.01,0.01) | -0.01(-0.01,-0.00) | 0.00(-0.01,0.01) | -0.00(-0.02,0.01) | 0.00(-0.02,0.02) |
| Coronavirus test | 0.21(0.13,0.28)* | -0.00(-0.04,0.04) | 0.13(0.05,0.21)* | 0.14(0.06,0.23)* | 0.25(0.13,0.36)* | 0.35(0.18,0.52)* |
| Household physical activity: Less | 0.54(0.46,0.62)* | 0.00(-0.04,0.04) | 0.39(0.30,0.49)* | 0.64(0.53,0.75)* | 0.80(0.67,0.93)* | 0.80(0.64,0.95)* |
| Household physical activity: More | 0.26(0.22,0.29)* | 0.00(-0.02,0.02) | 0.19(0.16,0.22)* | 0.29(0.25,0.33)* | 0.32(0.27,0.37)* | 0.35(0.27,0.42)* |
| Sport-related physical activity: Less | 0.21(0.18,0.24)* | 0.00(-0.02,0.02) | 0.14(0.12,0.16)* | 0.23(0.19,0.27)* | 0.24(0.19,0.29)* | 0.22(0.14,0.29)* |
| Sport-related physical activity: More | 0.15(0.10,0.19)* | 0.00(-0.02,0.02) | 0.08(0.05,0.11)* | 0.17(0.13,0.22)* | 0.18(0.12,0.24)* | 0.22(0.13,0.31)* |
| Sitting behavior: Less | 0.27(0.21,0.34)* | 0.00(-0.02,0.02) | 0.08(0.03,0.12)* | 0.23(0.16,0.31)* | 0.35(0.25,0.45)* | 0.47(0.34,0.61)* |
| Sitting behavior: More | 0.23(0.19,0.26)* | 0.00(-0.02,0.02) | 0.18(0.15,0.21)* | 0.26(0.22,0.30)* | 0.27(0.22,0.32)* | 0.34(0.26,0.41)* |
| Smoking status: Past smoker | 0.03(0.00,0.07) | 0.00(-0.02,0.02) | 0.04(0.03,0.06)* | 0.06(0.03,0.10)* | 0.06(0.01,0.11) | 0.02(-0.05,0.09) |
| Smoking status: Current smoker | -0.05(-0.10,0.00) | -0.00(-0.02,0.02) | -0.03(-0.06,-0.00) | -0.02(-0.07,0.03) | -0.02(-0.09,0.05) | -0.01(-0.11,0.10) |
| Alcohol consumption: 2 to 4 times/month | 0.07(0.03,0.11)* | 0.00(-0.02,0.02) | 0.08(0.06,0.11)* | 0.10(0.05,0.14)* | 0.05(-0.01,0.11) | 0.03(-0.06,0.13) |
| Alcohol consumption: 2 to 4 times/week | 0.18(0.14,0.23)* | 0.00(-0.02,0.02) | 0.15(0.12,0.18)* | 0.21(0.17,0.26)* | 0.19(0.13,0.25)* | 0.13(0.04,0.21)* |
| Alcohol consumption: ≥ 5 times/week | 0.29(0.24,0.34)* | 0.00(-0.02,0.02) | 0.16(0.14,0.19)* | 0.25(0.20,0.31)* | 0.33(0.26,0.40)* | 0.30(0.19,0.42)* |
| Depression score: ≥10 | 4.43(4.37,4.49)* | 4.00(3.94,4.06)* | 3.61(3.52,3.69)* | 4.06(3.94,4.17)* | 5.08(4.94,5.23)* | 6.11(5.91,6.30)* |
| Stress score: ≥10 | 2.00(1.94,2.05)* | 2.00(1.96,2.04)* | 1.94(1.88,2.00)* | 2.04(1.96,2.11)* | 2.12(2.02,2.22)* | 2.28(2.14,2.42)* |
| Smoking change: Yes to No^b^ | 0.05(-0.03,0.12) | 0.00(-0.04,0.04) | 0.00(-0.05,0.05) | 0.05(-0.02,0.13) | 0.13(0.01,0.25) | 0.17(0.03,0.31) |
| Smoking change: No to Yes^b^ | 0.02(-0.09,0.14) | 0.00(-0.06,0.06) | 0.07(-0.05,0.18) | 0.11(-0.00,0.23) | 0.09(-0.09,0.28) | 0.04(-0.23,0.30) |
| Smoking change: Remained Yes^b^ | -0.05(-0.10,-0.01) | -0.00(-0.02,0.02) | -0.06(-0.09,-0.03)* | -0.05(-0.10,-0.00) | -0.03(-0.10,0.04) | -0.01(-0.11,0.10) |
| Alcohol consumption change: Less^b^ | -0.04(-0.08,0.00) | 0.00(-0.01,0.01) | -0.04(-0.06,-0.03)* | -0.05(-0.09,-0.01) | -0.01(-0.07,0.04) | 0.03(-0.05,0.10) |
| Alcohol consumption change: More^b^ | 0.18(0.14,0.22)* | 0.00(-0.02,0.02) | 0.09(0.06,0.12)* | 0.12(0.08,0.17)* | 0.22(0.16,0.27)* | 0.29(0.21,0.38)* |

Note: The table presents the absolute changes in anxiety change and 95% CI from the linear/quantile regression model.

The model included smoking, alcohol, and mental health scales during COVID-19 and was adjusted with anxiety score at baseline and time from baseline to the COVID-NAKO survey.

^b^The model included variables’ changes from baseline to COVID-NAKO survey. *Statistically significant after Bonferroni correction.

TABLE S6: Factors associated with stress score change from baseline examination to early COVID-19.

| Variables | Linear | Q10 | Q25 | Q50 | Q75 | Q90 |
| --- | --- | --- | --- | --- | --- | --- |
| Age at baseline(years) | -0.02(-0.02,-0.02)* | -0.01(-0.01,-0.01)* | -0.01(-0.02,-0.01)* | -0.02(-0.02,-0.02)* | -0.03(-0.03,-0.02)* | -0.03(-0.04,-0.03)* |
| Gender: Male | -0.40(-0.44,-0.37)* | -0.19(-0.22,-0.16)* | -0.29(-0.32,-0.25)* | -0.41(-0.45,-0.37)* | -0.50(-0.55,-0.44)* | -0.52(-0.60,-0.44)* |
| Education at baseline: University | -0.03(-0.06,0.01) | 0.10(0.08,0.13)* | 0.11(0.07,0.14)* | 0.02(-0.02,0.07) | -0.11(-0.17,-0.05)* | -0.30(-0.38,-0.22)* |
| Employment status: Employed | 0.49(0.44,0.53)* | 0.14(0.11,0.17)* | 0.26(0.22,0.30)* | 0.40(0.35,0.45)* | 0.64(0.57,0.70)* | 0.85(0.75,0.95)* |
| Cohabitation: Living with others | 0.29(0.24,0.33)* | 0.11(0.07,0.14)* | 0.19(0.14,0.23)* | 0.30(0.24,0.35)* | 0.34(0.26,0.41)* | 0.37(0.27,0.47)* |
| Financial status: Deteriorated | 0.99(0.95,1.03)* | 0.51(0.46,0.56)* | 0.75(0.70,0.80)* | 0.99(0.93,1.05)* | 1.21(1.13,1.29)* | 1.38(1.27,1.48)* |
| Financial status: Improved | -0.13(-0.23,-0.04)* | 0.05(-0.03,0.13) | 0.03(-0.04,0.10) | -0.08(-0.18,0.02) | -0.24(-0.37,-0.11)* | -0.33(-0.53,-0.13)* |
| Self-rated health status | 0.76(0.73,0.78)* | 0.42(0.40,0.44)* | 0.60(0.57,0.62)* | 0.75(0.72,0.78)* | 0.90(0.86,0.94)* | 0.98(0.93,1.04)* |
| Life satisfaction at baseline | -0.01(-0.02,0.00) | -0.00(-0.01,0.01) | -0.01(-0.02,0.01) | -0.02(-0.03,-0.00) | -0.02(-0.04,-0.00) | -0.04(-0.06,-0.02)* |
| Coronavirus test | 0.36(0.28,0.44)* | 0.26(0.17,0.35)* | 0.38(0.29,0.47)* | 0.34(0.25,0.44)* | 0.40(0.28,0.52)* | 0.47(0.32,0.63)* |
| Household physical activity: Less | 0.82(0.73,0.90)* | 0.57(0.48,0.66)* | 0.77(0.67,0.87)* | 0.82(0.71,0.93)* | 0.98(0.83,1.13)* | 1.01(0.82,1.21)* |
| Household physical activity: More | 0.34(0.30,0.38)* | 0.24(0.21,0.27)* | 0.29(0.25,0.34)* | 0.35(0.30,0.40)* | 0.40(0.34,0.46)* | 0.40(0.31,0.48)* |
| Sport-related physical activity: Less | 0.53(0.49,0.57)* | 0.31(0.28,0.34)* | 0.39(0.35,0.43)* | 0.53(0.48,0.57)* | 0.61(0.55,0.67)* | 0.67(0.59,0.76)* |
| Sport-related physical activity: More | 0.10(0.05,0.15)* | 0.07(0.03,0.10)* | 0.09(0.04,0.13)* | 0.12(0.06,0.17)* | 0.11(0.03,0.19)* | 0.15(0.04,0.25)* |
| Sitting behavior: Less | 0.36(0.29,0.42)* | 0.06(-0.01,0.13) | 0.20(0.12,0.27)* | 0.36(0.27,0.44)* | 0.49(0.38,0.60)* | 0.59(0.43,0.75)* |
| Sitting behavior: More | 0.42(0.38,0.46)* | 0.33(0.29,0.37)* | 0.44(0.40,0.48)* | 0.46(0.41,0.51)* | 0.44(0.38,0.51)* | 0.43(0.35,0.52)* |
| Smoking status: Past smoker | 0.10(0.06,0.14)* | 0.04(0.01,0.07)* | 0.06(0.02,0.10)* | 0.14(0.09,0.19)* | 0.15(0.09,0.21)* | 0.16(0.07,0.24)* |
| Smoking status: Current smoker | 0.00(-0.05,0.05) | -0.06(-0.10,-0.02)* | -0.05(-0.11,0.01) | 0.02(-0.05,0.08) | 0.05(-0.03,0.14) | 0.04(-0.07,0.16) |
| Alcohol consumption: 2 to 4 times/month | 0.02(-0.02,0.07) | 0.03(-0.01,0.07) | 0.02(-0.03,0.07) | 0.05(-0.00,0.11) | -0.01(-0.09,0.07) | -0.05(-0.16,0.06) |
| Alcohol consumption: 2 to 4 times/week | 0.04(-0.01,0.08) | 0.04(0.01,0.08) | 0.07(0.03,0.12)* | 0.08(0.02,0.13)* | 0.00(-0.07,0.08) | -0.09(-0.19,0.02) |
| Alcohol consumption: ≥ 5 times/week | 0.12(0.07,0.18)* | 0.10(0.05,0.14)* | 0.12(0.07,0.17)* | 0.14(0.08,0.20)* | 0.08(-0.00,0.17) | -0.02(-0.14,0.10) |
| Depression score: ≥10 | 1.94(1.87,2.02)* | 1.74(1.65,1.84)* | 1.93(1.82,2.03)* | 2.05(1.94,2.16)* | 2.12(1.98,2.25)* | 2.02(1.88,2.17)* |
| Anxiety score: ≥10 | 1.96(1.87,2.05)* | 2.03(1.87,2.19)* | 2.05(1.93,2.16)* | 2.00(1.86,2.13)* | 2.00(1.83,2.16)* | 2.08(1.92,2.25)* |
| Smoking change: Yes to No^b^ | 0.02(-0.06,0.11) | -0.01(-0.08,0.06) | 0.02(-0.08,0.12) | 0.09(0.00,0.19) | 0.05(-0.06,0.16) | 0.04(-0.13,0.21) |
| Smoking change: No to Yes^b^ | 0.07(-0.06,0.20) | -0.06(-0.22,0.09) | -0.06(-0.21,0.10) | 0.08(-0.12,0.28) | 0.25(-0.04,0.53) | 0.35(0.07,0.62) |
| Smoking change: Remained Yes^b^ | -0.05(-0.10,0.00) | -0.08(-0.11,-0.04)* | -0.07(-0.14,-0.01) | -0.03(-0.09,0.03) | -0.05(-0.14,0.04) | -0.06(-0.19,0.06) |
| Alcohol consumption change: Less^b^ | -0.01(-0.05,0.04) | -0.04(-0.06,-0.01) | -0.04(-0.08,-0.00) | -0.01(-0.06,0.04) | 0.04(-0.02,0.11) | 0.04(-0.05,0.14) |
| Alcohol consumption change: More^b^ | 0.23(0.19,0.28)* | 0.09(0.06,0.12)* | 0.15(0.10,0.20)* | 0.24(0.19,0.30)* | 0.30(0.23,0.37)* | 0.32(0.22,0.42)* |

Note: The table presents the absolute changes in stress change and 95% CI from the linear/quantile regression model.

The model included smoking, alcohol, and mental health scales during the COVID-19 and was adjusted with stress score at baseline and time from baseline to the COVID-NAKO survey

^b^The model included variables’ changes from baseline to COVID-NAKO survey. *Statistically significant after Bonferroni correction.

TABLE S7. Factors associated with self-rated health change from baseline to early COVID-19, adjusted for baseline years.

| Variables | Better | Worse |
| --- | --- | --- |
| Age at baseline (years) | 0.97(0.97,0.97) | 1.02(1.01,1.02) |
| Gender: Male | 1.14(1.11,1.18) | 0.90(0.86,0.94) |
| Education at baseline: University | 1.11(1.08,1.15) | 0.85(0.81,0.89) |
| Employment status: Employed | 1.10(1.05,1.14) | 0.88(0.83,0.93) |
| Cohabitation: Living with others | 0.96(0.92,1.00) | 0.99(0.93,1.05) |
| Financial status: Deteriorated | 1.09(1.05,1.13) | 0.95(0.90,1.01) |
| Financial status: Improved | 1.07(0.98,1.16) | 0.94(0.83,1.06) |
| Life satisfaction at baseline | 1.12(1.11,1.13) | 0.94(0.93,0.95) |
| Coronavirus test | 0.78(0.72,0.84) | 1.60(1.46,1.75) |
| Household physical activity: Less | 0.85(0.78,0.92) | 1.44(1.31,1.59) |
| Household physical activity: More | 1.05(1.01,1.09) | 0.97(0.93,1.02) |
| Sport-related physical activity: Less | 0.96(0.93,1.00) | 1.14(1.09,1.20) |
| Sport-related physical activity: More | 1.37(1.31,1.43) | 0.78(0.73,0.83) |
| Sitting behavior: Less | 1.02(0.96,1.09) | 1.11(1.02,1.21) |
| Sitting behavior: More | 0.90(0.87,0.93) | 1.13(1.07,1.19) |
| Smoking status: Past smoker | 0.96(0.93,1.00) | 1.14(1.09,1.20) |
| Smoking status: Current smoker | 0.90(0.86,0.94) | 1.23(1.14,1.31) |
| Alcohol consumption: 2 to 4 times/month | 1.03(0.98,1.07) | 0.86(0.81,0.92) |
| Alcohol consumption: 2 to 4 times/week | 1.11(1.06,1.15) | 0.78(0.73,0.83) |
| Alcohol consumption: ≥ 5 times/week | 1.12(1.07,1.18) | 0.82(0.77,0.88) |
| Depression score: ≥10 | 0.53(0.50,0.57) | 2.32(2.13,2.52) |
| Anxiety score: ≥10 | 0.77(0.71,0.84) | 1.64(1.49,1.81) |
| Stress score: ≥10 | 0.64(0.60,0.68) | 1.78(1.66,1.92) |
| Smoking change: Yes to No^b^ | 0.98(0.91,1.05) | 1.22(1.10,1.35) |
| Smoking change: No to Yes^b^ | 1.00(0.89,1.12) | 1.18(1.01,1.38) |
| Smoking change: Remained Yes^b^ | 0.90(0.86,0.95) | 1.17(1.09,1.25) |
| Alcohol consumption change: Less^b^ | 0.96(0.92,0.99) | 1.15(1.09,1.21) |
| Alcohol consumption change: More^b^ | 1.00(0.96,1.04) | 0.93(0.88,0.98) |
| Depression change: Decreased from ≥10 to <10^b^ | 1.02(0.93,1.12) | 1.14(0.96,1.34) |
| Depression change: Increased from <10 to ≥10^b^ | 0.53(0.48,0.57) | 2.42(2.21,2.65) |
| Depression change: Remained ≥10^b^ | 0.53(0.47,0.60) | 2.08(1.76,2.45) |
| Anxiety change: Decreased from ≥10 to <10^b^ | 1.24(1.11,1.38) | 0.98(0.81,1.18) |
| Anxiety change: Increased from <10 to ≥10^b^ | 0.79(0.71,0.87) | 1.66(1.50,1.84) |
| Anxiety change: Remained ≥10^b^ | 0.81(0.69,0.95) | 1.63(1.34,1.99) |
| Stress change: Decreased from ≥10 to <10^b^ | 0.86(0.78,0.96) | 1.21(1.00,1.46) |
| Stress change: Increased from <10 to ≥10^b^ | 0.62(0.58,0.66) | 1.83(1.69,1.97) |
| Stress change: Remained ≥10^b^ | 0.66(0.59,0.75) | 1.70(1.44,2.01) |
| Baseline year: 2015 | 1.03(0.92,1.15) | 1.12(0.96,1.30) |
| Baseline year: 2016 | 1.02(0.89,1.17) | 1.19(0.98,1.45) |
| Baseline year: 2017 | 1.01(0.84,1.21) | 1.18(0.91,1.53) |
| Baseline year: 2018 | 1.03(0.81,1.30) | 1.23(0.88,1.72) |
| Baseline year: 2019 | 1.14(0.87,1.51) | 1.28(0.86,1.89) |

Note: The table presents the Relative Risk Ratio (RRR) and 95% confidence interval (CI) from the multinomial logistic regression model, using “Same” as the reference group.

The model included smoking, alcohol, and mental health scales during COVID-19 and was adjusted with health status at baseline and time from baseline to the COVID-NAKO survey.

^b^The model included variables’ changes from baseline to the COVID-NAKO survey.

TABLE S8. Factors associated with self-rated health change from baseline to early COVID-19, adjusted for the COVID-NAKO survey date.

| Variables | Better | Worse |
| --- | --- | --- |
| Age at baseline (years) | 0.97(0.97,0.97) | 1.02(1.01,1.02) |
| Gender: Male | 1.15(1.15,1.15) | 0.90(0.90,0.90) |
| Education at baseline: University | 1.11(1.11,1.11) | 0.85(0.85,0.85) |
| Employment status: Employed | 1.10(1.10,1.10) | 0.88(0.88,0.88) |
| Cohabitation: Living with others | 0.96(0.96,0.96) | 0.99(0.99,0.99) |
| Financial status: Deteriorated | 1.09(1.09,1.09) | 0.95(0.95,0.95) |
| Financial status: Improved | 1.07(1.07,1.07) | 0.94(0.94,0.94) |
| Life satisfaction at baseline | 1.12(1.12,1.12) | 0.94(0.94,0.94) |
| Coronavirus test | 0.78(0.78,0.78) | 1.59(1.59,1.59) |
| Household physical activity: Less | 0.85(0.85,0.85) | 1.44(1.44,1.44) |
| Household physical activity: More | 1.05(1.05,1.05) | 0.98(0.98,0.98) |
| Sport-related physical activity: Less | 0.96(0.96,0.96) | 1.15(1.15,1.15) |
| Sport-related physical activity: More | 1.37(1.37,1.37) | 0.78(0.78,0.78) |
| Sitting behavior: Less | 1.02(1.02,1.02) | 1.12(1.12,1.12) |
| Sitting behavior: More | 0.89(0.89,0.89) | 1.14(1.14,1.14) |
| Smoking status: Past smoker | 0.96(0.96,0.96) | 1.14(1.14,1.14) |
| Smoking status: Current smoker | 0.90(0.90,0.90) | 1.22(1.22,1.22) |
| Alcohol consumption: 2 to 4 times/month | 1.03(1.03,1.03) | 0.86(0.86,0.86) |
| Alcohol consumption: 2 to 4 times/week | 1.11(1.11,1.11) | 0.78(0.78,0.78) |
| Alcohol consumption: ≥ 5 times/week | 1.12(1.12,1.12) | 0.82(0.82,0.82) |
| Depression score: ≥10 | 0.53(0.53,0.53) | 2.33(2.33,2.33) |
| Anxiety score: ≥10 | 0.77(0.77,0.77) | 1.64(1.64,1.64) |
| Stress score: ≥10 | 0.64(0.64,0.64) | 1.78(1.78,1.78) |
| Smoking change: Yes to No^b^ | 0.98(0.98,0.98) | 1.22(1.22,1.22) |
| Smoking change: No to Yes^b^ | 1.00(1.00,1.00) | 1.18(1.18,1.18) |
| Smoking change: Remained Yes^b^ | 0.90(0.90,0.90) | 1.16(1.16,1.16) |
| Alcohol consumption change: Less^b^ | 0.96(0.96,0.96) | 1.15(1.15,1.15) |
| Alcohol consumption change: More^b^ | 1.00(1.00,1.00) | 0.93(0.93,0.93) |
| Depression change: Decreased from ≥10 to <10^b^ | 1.02(1.02,1.02) | 1.14(1.14,1.14) |
| Depression change: Increased from <10 to ≥10^b^ | 0.52(0.52,0.52) | 2.44(2.44,2.44) |
| Depression change: Remained ≥10^b^ | 0.53(0.53,0.53) | 2.09(2.09,2.09) |
| Anxiety change: Decreased from ≥10 to <10^b^ | 1.24(1.24,1.24) | 0.99(0.99,0.99) |
| Anxiety change: Increased from <10 to ≥10^b^ | 0.78(0.78,0.78) | 1.66(1.66,1.66) |
| Anxiety change: Remained ≥10^b^ | 0.81(0.81,0.81) | 1.64(1.64,1.64) |
| Stress change: Decreased from ≥10 to <10^b^ | 0.87(0.87,0.87) | 1.20(1.20,1.20) |
| Stress change: Increased from <10 to ≥10^b^ | 0.62(0.62,0.62) | 1.83(1.83,1.83) |
| Stress change: Remained ≥10^b^ | 0.67(0.67,0.67) | 1.70(1.70,1.70) |
| Date: COVID-NAKO | 0.98(0.98,0.98) | 1.02(1.02,1.02) |

Note: The table presents the Relative Risk Ratio (RRR) and 95% confidence interval (CI) from the multinomial logistic regression model, using “Same” as the reference group.

The model included smoking, alcohol, and mental health scales during COVID-19 and was adjusted with health status at baseline and time from baseline to the COVID-NAKO survey.

^b^The model included variables’ changes from baseline to the COVID-NAKO survey.

TABLE S9. Factors associated with self-rated health change from baseline to early COVID-19, adjusted for the study centers.

| Variables | Better | Worse |
| --- | --- | --- |
| Age at baseline (years) | 0.97(0.97,0.97) | 1.02(1.01,1.02) |
| Gender: Male | 1.15(1.11,1.19) | 0.90(0.86,0.94) |
| Education at baseline: University | 1.11(1.07,1.15) | 0.85(0.81,0.89) |
| Employment status: Employed | 1.10(1.05,1.14) | 0.88(0.83,0.93) |
| Cohabitation: Living with others | 0.96(0.92,1.00) | 0.99(0.93,1.05) |
| Financial status: Deteriorated | 1.09(1.04,1.13) | 0.96(0.90,1.01) |
| Financial status: Improved | 1.07(0.98,1.16) | 0.94(0.83,1.06) |
| Life satisfaction at baseline | 1.12(1.11,1.13) | 0.94(0.93,0.95) |
| Coronavirus test | 0.77(0.72,0.83) | 1.60(1.46,1.75) |
| Household physical activity: Less | 0.85(0.78,0.92) | 1.44(1.31,1.59) |
| Household physical activity: More | 1.05(1.01,1.08) | 0.97(0.93,1.02) |
| Sport-related physical activity: Less | 0.96(0.93,1.00) | 1.14(1.09,1.20) |
| Sport-related physical activity: More | 1.37(1.31,1.43) | 0.78(0.73,0.83) |
| Sitting behavior: Less | 1.03(0.96,1.09) | 1.11(1.02,1.21) |
| Sitting behavior: More | 0.90(0.86,0.93) | 1.13(1.07,1.19) |
| Smoking status: Past smoker | 0.96(0.93,1.00) | 1.14(1.08,1.20) |
| Smoking status: Current smoker | 0.90(0.85,0.94) | 1.23(1.14,1.31) |
| Alcohol consumption: 2 to 4 times/month | 1.02(0.98,1.07) | 0.86(0.81,0.92) |
| Alcohol consumption: 2 to 4 times/week | 1.10(1.06,1.15) | 0.78(0.73,0.83) |
| Alcohol consumption: ≥ 5 times/week | 1.11(1.06,1.17) | 0.83(0.77,0.89) |
| Depression score: ≥10 | 0.53(0.49,0.57) | 2.32(2.13,2.53) |
| Anxiety score: ≥10 | 0.77(0.71,0.84) | 1.65(1.49,1.82) |
| Stress score: ≥10 | 0.64(0.60,0.68) | 1.78(1.65,1.91) |
| Smoking change: Yes to No^b^ | 0.97(0.90,1.05) | 1.23(1.11,1.37) |
| Smoking change: No to Yes^b^ | 0.99(0.88,1.12) | 1.17(1.00,1.37) |
| Smoking change: Remained Yes^b^ | 0.90(0.86,0.95) | 1.17(1.09,1.26) |
| Alcohol consumption change: Less^b^ | 0.96(0.92,0.99) | 1.15(1.09,1.21) |
| Alcohol consumption change: More^b^ | 1.00(0.96,1.04) | 0.93(0.88,0.98) |
| Depression change: Decreased from ≥10 to <10^b^ | 1.02(0.93,1.12) | 1.13(0.96,1.34) |
| Depression change: Increased from <10 to ≥10^b^ | 0.52(0.48,0.57) | 2.41(2.20,2.64) |
| Depression change: Remained ≥10^b^ | 0.53(0.47,0.60) | 2.08(1.76,2.45) |
| Anxiety change: Decreased from ≥10 to <10^b^ | 1.24(1.11,1.38) | 0.98(0.81,1.19) |
| Anxiety change: Increased from <10 to ≥10^b^ | 0.79(0.71,0.87) | 1.66(1.50,1.85) |
| Anxiety change: Remained ≥10^b^ | 0.81(0.69,0.95) | 1.63(1.34,1.99) |
| Stress change: Decreased from ≥10 to <10^b^ | 0.86(0.78,0.96) | 1.21(1.00,1.46) |
| Stress change: Increased from <10 to ≥10^b^ | 0.62(0.58,0.66) | 1.83(1.70,1.98) |
| Stress change: Remained ≥10^b^ | 0.67(0.59,0.75) | 1.70(1.44,2.01) |
| Regensburg | 0.94(0.87,1.02) | 1.05(0.94,1.18) |
| Mannheim | 0.99(0.91,1.08) | 1.02(0.90,1.15) |
| Freiburg | 0.97(0.89,1.05) | 1.02(0.91,1.14) |
| Saarbrücken | 0.89(0.82,0.97) | 1.11(0.99,1.25) |
| Essen | 0.92(0.85,1.00) | 1.10(0.97,1.24) |
| Münster | 0.83(0.77,0.91) | 1.15(1.03,1.29) |
| Düsseldorf | 1.01(0.93,1.11) | 0.92(0.81,1.05) |
| Halle | 0.98(0.90,1.07) | 1.10(0.97,1.24) |
| Leipzig | 0.94(0.86,1.02) | 0.96(0.85,1.09) |
| Berlin-Nord | 0.93(0.86,1.01) | 1.15(1.03,1.29) |
| Berlin-Mitte | 0.97(0.89,1.05) | 1.04(0.93,1.17) |
| Berlin-Süd | 1.03(0.94,1.11) | 1.13(1.01,1.27) |
| Hannover | 0.85(0.78,0.93) | 1.22(1.08,1.38) |
| Hamburg | 1.05(0.96,1.14) | 0.97(0.85,1.09) |
| Bremen | 0.95(0.87,1.03) | 1.05(0.94,1.18) |
| Kiel | 0.90(0.83,0.98) | 1.11(0.99,1.25) |
| Neubrandenburg | 0.85(0.79,0.92) | 1.05(0.94,1.18) |

Note: The table presents the Relative Risk Ratio (RRR) and 95% confidence interval (CI) from the multinomial logistic regression model, using “Same” as the reference group.

The model included smoking, alcohol, and mental health scales during COVID-19 and was adjusted with health status at baseline and time from baseline to the COVID-NAKO survey.

^b^The model included variables’ changes from baseline to the COVID-NAKO survey.

TABLE S10. Factors associated with self-rated health change from baseline to early COVID-19, with inverse probability weighting.

| Variables | Better | Worse |
| --- | --- | --- |
| Age at baseline (years) | 0.97(0.97,0.97) | 1.02(1.01,1.02) |
| Gender: Male | 1.14(1.10,1.18) | 0.91(0.87,0.95) |
| Education at baseline: University | 1.11(1.07,1.15) | 0.85(0.81,0.89) |
| Employment status: Employed | 1.10(1.06,1.14) | 0.88(0.83,0.93) |
| Cohabitation: Living with others | 0.95(0.91,0.99) | 1.00(0.94,1.06) |
| Financial status: Deteriorated | 1.09(1.05,1.13) | 0.95(0.90,1.01) |
| Financial status: Improved | 1.07(0.98,1.17) | 0.94(0.83,1.06) |
| Life satisfaction at baseline | 1.12(1.11,1.13) | 0.94(0.93,0.95) |
| Coronavirus test | 0.77(0.71,0.83) | 1.62(1.47,1.77) |
| Household physical activity: Less | 0.85(0.78,0.92) | 1.44(1.31,1.59) |
| Household physical activity: More | 1.05(1.01,1.09) | 0.97(0.92,1.02) |
| Sport-related physical activity: Less | 0.96(0.93,1.00) | 1.15(1.09,1.20) |
| Sport-related physical activity: More | 1.38(1.32,1.44) | 0.78(0.73,0.83) |
| Sitting behavior: Less | 1.02(0.96,1.08) | 1.11(1.02,1.21) |
| Sitting behavior: More | 0.90(0.87,0.93) | 1.13(1.08,1.19) |
| Smoking status: Past smoker | 0.96(0.93,1.00) | 1.14(1.09,1.20) |
| Smoking status: Current smoker | 0.90(0.86,0.94) | 1.23(1.14,1.31) |
| Alcohol consumption: 2 to 4 times/month | 1.03(0.99,1.08) | 0.87(0.82,0.92) |
| Alcohol consumption: 2 to 4 times/week | 1.11(1.07,1.16) | 0.78(0.74,0.83) |
| Alcohol consumption: ≥ 5 times/week | 1.13(1.07,1.19) | 0.82(0.77,0.88) |
| Depression score: ≥10 | 0.53(0.49,0.57) | 2.35(2.15,2.56) |
| Anxiety score: ≥10 | 0.78(0.71,0.85) | 1.63(1.47,1.80) |
| Stress score: ≥10 | 0.64(0.60,0.68) | 1.79(1.66,1.93) |
| Smoking change: Yes to No^b^ | 0.97(0.90,1.04) | 1.24(1.12,1.38) |
| Smoking change: No to Yes^b^ | 1.00(0.89,1.13) | 1.17(1.00,1.38) |
| Smoking change: Remained Yes^b^ | 0.91(0.86,0.95) | 1.17(1.09,1.25) |
| Alcohol consumption change: Less^b^ | 0.95(0.92,0.99) | 1.15(1.09,1.21) |
| Alcohol consumption change: More^b^ | 1.00(0.96,1.04) | 0.93(0.88,0.99) |
| Depression change: Decreased from ≥10 to <10^b^ | 1.02(0.93,1.12) | 1.16(0.98,1.37) |
| Depression change: Increased from <10 to ≥10^b^ | 0.52(0.48,0.57) | 2.45(2.24,2.68) |
| Depression change: Remained ≥10^b^ | 0.53(0.47,0.60) | 2.13(1.80,2.51) |
| Anxiety change: Decreased from ≥10 to <10^b^ | 1.24(1.12,1.38) | 1.00(0.83,1.21) |
| Anxiety change: Increased from <10 to ≥10^b^ | 0.79(0.71,0.88) | 1.65(1.48,1.83) |
| Anxiety change: Remained ≥10^b^ | 0.81(0.69,0.96) | 1.62(1.32,1.97) |
| Stress change: Decreased from ≥10 to <10^b^ | 0.87(0.78,0.97) | 1.21(1.00,1.46) |
| Stress change: Increased from <10 to ≥10^b^ | 0.62(0.58,0.66) | 1.83(1.70,1.98) |
| Stress change: Remained ≥10^b^ | 0.67(0.59,0.76) | 1.67(1.41,1.97) |

Note: The table presents the Relative Risk Ratio (RRR) and 95% confidence interval (CI) from the multinomial logistic regression model, using “Same” as the reference group.

The model included smoking, alcohol, and mental health scales during COVID-19 and was adjusted with health status at baseline and time from baseline to the COVID-NAKO survey.

^b^The model included variables’ changes from baseline to the COVID-NAKO survey.

TABLE S11. Factors associated with depression score during early COVID-19, adjusted for baseline years.

| Variables | Linear |
| --- | --- |
| Age at baseline (years) | -0.04 (-0.05, -0.04) |
| Gender: Male | -0.33 (-0.36, -0.29) |
| Education at baseline: University | 0.16 (0.12, 0.19) |
| Employment status: Employed | -0.10 (-0.15, -0.06) |
| Cohabitation: Living with others | -0.44 (-0.49, -0.40) |
| Financial status: Deteriorated | 0.51 (0.47, 0.56) |
| Financial status: Improved | -0.10 (-0.20, -0.01) |
| Self-rated health status | 0.84 (0.81, 0.87) |
| Life satisfaction at baseline | -0.04 (-0.05, -0.03) |
| Coronavirus test | -0.01 (-0.09, 0.07) |
| Household physical activity: Less | 1.49 (1.40, 1.57) |
| Household physical activity: More | 0.23 (0.19, 0.27) |
| Sport-related physical activity: Less | 0.39 (0.35, 0.43) |
| Sport-related physical activity: More | 0.12 (0.07, 0.17) |
| Sitting behavior: Less | 0.23 (0.16, 0.30) |
| Sitting behavior: More | 0.49 (0.45, 0.53) |
| Smoking status: Past smoker | 0.11 (0.07, 0.14) |
| Smoking status: Current smoker | 0.11 (0.06, 0.17) |
| Alcohol consumption: 2 to 4 times/month | 0.00 (-0.04, 0.05) |
| Alcohol consumption: 2 to 4 times/week | 0.10 (0.05, 0.14) |
| Alcohol consumption: ≥ 5 times/week | 0.24 (0.18, 0.29) |
| Anxiety score: ≥10 | 5.80 (5.72, 5.88) |
| Stress score: ≥10 | 2.09 (2.03, 2.15) |
| Smoking change: Yes to No^b^ | 0.11 (0.02, 0.19) |
| Smoking change: No to Yes^b^ | 0.20 (0.07, 0.33) |
| Smoking change: Remained Yes^b^ | 0.08 (0.02, 0.13) |
| Alcohol consumption change: Less^b^ | -0.05 (-0.10, -0.01) |
| Alcohol consumption change: More^b^ | 0.22 (0.17, 0.26) |
| Baseline year: 2015 | -0.09 (-0.21, 0.03) |
| Baseline year: 2016 | -0.05 (-0.20, 0.11) |
| Baseline year: 2017 | -0.18 (-0.38, 0.02) |
| Baseline year: 2018 | -0.13 (-0.39, 0.13) |
| Baseline year: 2019 | -0.13 (-0.43, 0.18) |

Note: The table presents the absolute changes in the depression scale and 95% CI from the linear/quantile regression model.

The model included smoking, alcohol, and mental health scales during COVID-19 and was adjusted with depression score at baseline and time from baseline to the COVID-NAKO survey.

^b^The model included variables’ changes from baseline to the COVID-NAKO survey.

TABLE S12. Factors associated with depression score during early COVID-19, adjusted for the COVID-NAKO survey date.

| Variables | Linear |
| --- | --- |
| Age at baseline (years) | -0.04 (-0.05, -0.04) |
| Gender: Male | -0.33 (-0.36, -0.29) |
| Education at baseline: University | 0.15 (0.12, 0.19) |
| Employment status: Employed | -0.10 (-0.15, -0.06) |
| Cohabitation: Living with others | -0.44 (-0.49, -0.40) |
| Financial status: Deteriorated | 0.51 (0.47, 0.56) |
| Financial status: Improved | -0.10 (-0.20, -0.01) |
| Self-rated health status | 0.84 (0.82, 0.87) |
| Life satisfaction at baseline | -0.04 (-0.05, -0.03) |
| Coronavirus test | -0.01 (-0.09, 0.07) |
| Household physical activity: Less | 1.49 (1.40, 1.57) |
| Household physical activity: More | 0.23 (0.19, 0.27) |
| Sport-related physical activity: Less | 0.39 (0.35, 0.42) |
| Sport-related physical activity: More | 0.12 (0.07, 0.17) |
| Sitting behavior: Less | 0.23 (0.16, 0.30) |
| Sitting behavior: More | 0.49 (0.45, 0.53) |
| Smoking status: Past smoker | 0.10 (0.07, 0.14) |
| Smoking status: Current smoker | 0.11 (0.06, 0.17) |
| Alcohol consumption: 2 to 4 times/month | 0.01 (-0.04, 0.05) |
| Alcohol consumption: 2 to 4 times/week | 0.10 (0.05, 0.14) |
| Alcohol consumption: ≥ 5 times/week | 0.24 (0.18, 0.29) |
| Anxiety score: ≥10 | 5.80 (5.71, 5.88) |
| Stress score: ≥10 | 2.09 (2.03, 2.15) |
| Smoking change: Yes to No^b^ | 0.11 (0.02, 0.19) |
| Smoking change: No to Yes^b^ | 0.20 (0.07, 0.33) |
| Smoking change: Remained Yes^b^ | 0.08 (0.03, 0.13) |
| Alcohol consumption change: Less^b^ | -0.06 (-0.10, -0.01) |
| Alcohol consumption change: More^b^ | 0.22 (0.17, 0.26) |
| Date: COVID-NAKO | -0.01 (-0.01, -0.00) |

Note: The table presents the absolute changes in the depression scale and 95% CI from the linear/quantile regression model.

The model included smoking, alcohol, and mental health scales during COVID-19 and was adjusted with depression score at baseline and time from baseline to the COVID-NAKO survey.

^b^The model included variables’ changes from baseline to the COVID-NAKO survey.

TABLE S13. Factors associated with depression score during early COVID-19, adjusted for the study centers.

| Variables | Linear |
| --- | --- |
| Age at baseline (years) | -0.05 (-0.05, -0.04) |
| Gender: Male | -0.33 (-0.36, -0.29) |
| Education at baseline: University | 0.13 (0.09, 0.17) |
| Employment status: Employed | -0.10 (-0.15, -0.06) |
| Cohabitation: Living with others | -0.43 (-0.48, -0.38) |
| Financial status: Deteriorated | 0.51 (0.47, 0.55) |
| Financial status: Improved | -0.11 (-0.20, -0.01) |
| Self-rated health status | 0.84 (0.82, 0.87) |
| Life satisfaction at baseline | -0.04 (-0.05, -0.03) |
| Coronavirus test | -0.01 (-0.09, 0.07) |
| Household physical activity: Less | 1.48 (1.39, 1.57) |
| Household physical activity: More | 0.22 (0.18, 0.26) |
| Sport-related physical activity: Less | 0.38 (0.34, 0.42) |
| Sport-related physical activity: More | 0.11 (0.06, 0.16) |
| Sitting behavior: Less | 0.23 (0.16, 0.30) |
| Sitting behavior: More | 0.48 (0.44, 0.52) |
| Smoking status: Past smoker | 0.10 (0.06, 0.14) |
| Smoking status: Current smoker | 0.11 (0.05, 0.16) |
| Alcohol consumption: 2 to 4 times/month | 0.01 (-0.04, 0.06) |
| Alcohol consumption: 2 to 4 times/week | 0.10 (0.05, 0.14) |
| Alcohol consumption: ≥ 5 times/week | 0.24 (0.18, 0.29) |
| Anxiety score: ≥10 | 5.80 (5.72, 5.88) |
| Stress score: ≥10 | 2.09 (2.03, 2.15) |
| Smoking change: Yes to No^b^ | 0.10 (0.02, 0.18) |
| Smoking change: No to Yes^b^ | 0.19 (0.06, 0.32) |
| Smoking change: Remained Yes^b^ | 0.08 (0.02, 0.13) |
| Alcohol consumption change: Less^b^ | -0.06 (-0.10, -0.02) |
| Alcohol consumption change: More^b^ | 0.22 (0.17, 0.26) |
| Regensburg | -0.01 (-0.10, 0.07) |
| Mannheim | -0.02 (-0.11, 0.08) |
| Freiburg | 0.09 (-0.00, 0.17) |
| Saarbrücken | -0.02 (-0.11, 0.08) |
| Essen | 0.01 (-0.08, 0.11) |
| Münster | 0.01 (-0.08, 0.10) |
| Düsseldorf | 0.03 (-0.07, 0.12) |
| Halle | -0.02 (-0.11, 0.08) |
| Leipzig | -0.19 (-0.28, -0.10) |
| Berlin-Nord | -0.03 (-0.12, 0.06) |
| Berlin-Mitte | 0.21 (0.12, 0.30) |
| Berlin-Süd | 0.14 (0.05, 0.23) |
| Hannover | 0.06 (-0.04, 0.16) |
| Hamburg | 0.09 (-0.01, 0.18) |
| Bremen | 0.10 (0.01, 0.19) |
| Kiel | 0.04 (-0.06, 0.13) |
| Neubrandenburg | -0.15 (-0.23, -0.06) |

Note: The table presents the absolute changes in the depression scale and 95% CI from the linear/quantile regression model.

The model included smoking, alcohol, and mental health scales during COVID-19 and was adjusted with depression score at baseline and time from baseline to the COVID-NAKO survey.

^b^The model included variables’ changes from baseline to the COVID-NAKO survey.

TABLE S14. Factors associated with depression score during early COVID-19, with inverse probability weighting.

| Variables | Linear |
| --- | --- |
| Age at baseline (years) | -0.04 (-0.05, -0.04) |
| Gender: Male | -0.33 (-0.37, -0.30) |
| Education at baseline: University | 0.15 (0.12, 0.19) |
| Employment status: Employed | -0.08 (-0.13, -0.04) |
| Cohabitation: Living with others | -0.44 (-0.49, -0.40) |
| Financial status: Deteriorated | 0.52 (0.47, 0.56) |
| Financial status: Improved | -0.11 (-0.20, -0.01) |
| Self-rated health status | 0.84 (0.81, 0.86) |
| Life satisfaction at baseline | -0.04 (-0.05, -0.03) |
| Coronavirus test | -0.01 (-0.09, 0.08) |
| Household physical activity: Less | 1.51 (1.42, 1.59) |
| Household physical activity: More | 0.23 (0.19, 0.27) |
| Sport-related physical activity: Less | 0.38 (0.34, 0.42) |
| Sport-related physical activity: More | 0.12 (0.07, 0.17) |
| Sitting behavior: Less | 0.24 (0.17, 0.31) |
| Sitting behavior: More | 0.49 (0.45, 0.53) |
| Smoking status: Past smoker | 0.11 (0.07, 0.15) |
| Smoking status: Current smoker | 0.11 (0.06, 0.16) |
| Alcohol consumption: 2 to 4 times/month | -0.00 (-0.05, 0.05) |
| Alcohol consumption: 2 to 4 times/week | 0.09 (0.05, 0.14) |
| Alcohol consumption: ≥ 5 times/week | 0.23 (0.17, 0.28) |
| Anxiety score: ≥10 | 5.81 (5.73, 5.90) |
| Stress score: ≥10 | 2.08 (2.02, 2.15) |
| Smoking change: Yes to No^b^ | 0.11 (0.03, 0.19) |
| Smoking change: No to Yes^b^ | 0.18 (0.05, 0.31) |
| Smoking change: Remained Yes^b^ | 0.07 (0.02, 0.13) |
| Alcohol consumption change: Less^b^ | -0.05 (-0.09, -0.01) |
| Alcohol consumption change: More^b^ | 0.21 (0.17, 0.26) |

Note: The table presents the absolute changes in the depression scale and 95% CI from the linear/quantile regression model.

The model included smoking, alcohol, and mental health scales during COVID-19 and was adjusted with depression score at baseline and time from baseline to the COVID-NAKO survey.

^b^The model included variables’ changes from baseline to the COVID-NAKO survey.
